# A bovine CD18 signal peptide variant with increased binding activity to *Mannheimia hemolytica* leukotoxin

**DOI:** 10.12688/f1000research.17187.1

**Published:** 2018-12-28

**Authors:** Aspen M. Workman, Carol G. Chitko-McKown, Timothy P. L. Smith, Gary L. Bennett, Theodore S. Kalbfleisch, Veronica Basnayake, Michael P. Heaton

**Affiliations:** 1 USDA, US Meat Animal Research Center (USMARC), Clay Center, Nebraska, 68933, USA; 2 Department of Biochemistry and Molecular Genetics, School of Medicine, University of Louisville, Louisville, Kentucky, 40292, USA; 3 GeneSeek, a Neogen Company, Lincoln, NE, USA

**Keywords:** Cattle, CD18, integrin beta 2, missense mutation, signal peptide variants, bacterial leukotoxin, bovine respiratory disease, shipping fever, Mannheimia haemolytica

## Abstract

**Background:** *Mannheimia haemolytica* is the major bacterial infectious agent of bovine respiratory disease complex and causes severe morbidity and mortality during lung infections. *M. haemolytica* secretes a protein leukotoxin (Lkt) that binds to the CD18 receptor on leukocytes, initiates lysis, induces inflammation, and causes acute fibrinous bronchopneumonia. Lkt binds the 22-amino acid CD18 signal peptide domain, which remains uncleaved in ruminant species. Our aim was to identify missense variation in the bovine CD18 signal peptide and measure the effects on Lkt binding.

**Methods:** Missense variants in the integrin beta 2 gene ( *ITGB2*) encoding CD18 were identified by whole genome sequencing of 96 cattle from 19 breeds, and targeted Sanger sequencing of 1238 cattle from 46 breeds. The ability of different CD18 signal peptide variants to bind Lkt was evaluated by preincubating the toxin with synthetic peptides and applying the mixture to susceptible bovine cell cultures in cytotoxicity-blocking assays.

**Results: **We identified 14 missense variants encoded on 15 predicted haplotypes, including a rare signal peptide variant with a cysteine at position 5 (C _5_) instead of arginine (R _5_). Preincubating Lkt with synthetic signal peptides with C _5_ blocked cytotoxicity significantly better than those with R _5_. The most potent synthetic peptide (C _5_PQLLLLAGLLA) had 30-fold more binding activity compared to that with R _5_.

**Conclusions: **The results suggest that missense variants in the CD18 signal peptide affect Lkt binding, and animals carrying the C _5_ allele may be more susceptible to the effects of Lkt. The results also identify a potent class of non-antibiotic Lkt inhibitors that could potentially protect cattle from cytotoxic effects during acute lung infections.

## Introduction

*Mannheimia haemolytica* is the major bacteria associated with bovine respiratory disease, a heterogeneous complex of highly infectious pathogens that are the primary cause of morbidity, mortality, and economic loss affecting beef and dairy cattle industries ^1, 2^. *M. haemolytica* is a commensal bacterium found in tonsillar crypts and the upper respiratory tracts of healthy cattle ^3, 4^. Exposure to environmental stresses or co-infection with other viral or bacterial pathogens can impair host defenses allowing *M. haemolytica* to proliferate and colonize the lungs where infection causes acute fibrinonecrotic pleuropneumonia ^2, 5, 6^. This bacterium expresses a variety of virulence factors that contribute to disease pathogenesis in the lungs. However, leukotoxin (Lkt) is the primary virulence factor contributing to the clinical signs and severe lung damage observed following infection ^7, 8^. Within hours of bacterial colonization of the lung, large numbers of polymorphonuclear leukocytes (PMN) infiltrate the airways ^2, 9^. Lkt binds to the bovine CD18 subunit of the heterodimeric integrins on the surface of PMN causing cell lysis and the release of pro-inflammatory cytokines, proteolytic enzymes, and reactive oxygen intermediates that intensify local inflammation ^6, 10– 13^. Experimental depletion of PMN prior to infection ^14^, or infection with a Lkt-deletion mutant of *M. haemolytica* ^7, 8^, results in decreased morbidity and reduced lung lesions in calves. Thus, the interaction between the toxin and its receptor is critical to the pathogenesis of *M. haemolytica* infection and is a potential intervention point for the prevention of disease.

A 13-amino acid sequence in the CD18 signal peptide has been identified as the site which binds to bacterial Lkt ^10^. The 22-amino acids that comprise the CD18 signal peptide remain uncleaved in leukocytes of ruminant species due to a conserved cleavage-inhibiting glutamine residue at position 18 (Q _18_) of the propeptide ^10^. In non-ruminant species, such as human and murine, leukocytes are naturally resistant to Lkt because their CD18 signal peptides undergo cleavage due to a glycine residue at position 18 (G _18_). However, when murine cell lines were transfected with bovine *ITGB2,* the gene which encodes CD18 *,* they became susceptible to Lkt. When site-directed mutagenesis of *ITGB2* was used in the same murine cell lines to change the bovine Q _18_ residue to G _18_, the bovine CD18 signal peptide was cleaved and the murine cells once again became resistant to Lkt-induced lysis ^10^. This strategy was taken further with gene-editing, showing that leukocytes isolated from a cloned bovine fetus, homozygous for CD18 G _18_, were unaffected by Lkt exposure because the signal peptide was cleaved and unavailable for Lkt binding ^15^. Thus, retention of the ruminant CD18 signal peptide appears to be the cause of Lkt sensitivity in leukocytes.

Naturally occurring CD18 amino acid sequence variation can also interfere with Lkt cytotoxicity. In Holstein dairy cattle, a CD18 substitution of glycine for aspartate at polypeptide position 128 in the extracellular I-like domain of CD18 causes bovine leukocyte adhesion deficiency (BLAD) in homozygous animals ^16, 17^. These calves do not express functional CD18 on the surface of their leukocytes and have significantly reduced sensitivity to Lkt compared to control calves ^18, 19^. We hypothesized that other variation in the CD18 polypeptide sequence, if it exists, may alter the Lkt-CD18 binding interaction or cell signaling and result in differences in lymphocyte sensitivity to *M. haemolytica* Lkt. Thus, the goals of this study were to identify CD18 protein variants encoded by *ITGB2* in U.S. cattle breeds, and evaluate the effects of signal peptide variants on Lkt binding. We report the identification of 15 predicted protein variants, including one with enhanced Lkt binding.

## Methods

### Ethics statement

All animal procedures were reviewed and approved by the U.S. Department of Agriculture, Agricultural Research Service, U.S. Meat Animal Research Center (USMARC) Institutional Animal Care and Use Committee (IACUC project number 2.2).

### Panels of cattle DNA used for missense mutation discovery

Two panels of DNAs were used to determine *ITGB2* genotypes from U.S. cattle. The first was a previously described panel of 96 unrelated beef cattle from 19 popular U.S. beef breeds that had already been characterized by whole-genome sequencing ^20^. This identified predicted coding changes throughout the *ITGB2* gene. The second panel included a non-overlapping set of 1142 unrelated cattle from 46 breeds, on which targeted Sanger sequencing was performed to identify any predicted coding changes in the signal peptide region and the region containing the D128G variant causing BLAD ( *ITGB2* exons 2, 3, and 5). Briefly, the first panel of 96 beef cattle (USMARC Beef Cattle Diversity Panel version 2.9 [MBCDPv2.9]) was based on commercially-available purebred registered sires. Pedigrees were obtained from leading suppliers of U.S. beef cattle semen and analyzed to identify unrelated individuals for inclusion. The number of sires representing each breed (four, five, or six) was based on their numbers of registered progeny circa 2000: Angus (n = 6), Hereford (n = 6), Charolais (n = 6), Simmental (n = 6), Red Angus (n = 6), Limousin (n = 6), Gelbvieh (n = 6), Brangus (n = 5), Beefmaster (n = 5), Salers (n = 5), Shorthorn (n = 5), Maine-Anjou (n = 5), Brahman (n = 5), Chianina (n = 4), Texas Longhorn (n = 4), Santa Gertrudis (n = 4), Braunvieh (n = 4), Corriente (n = 4), and Tarentaise (n = 4). On the basis of the number of registered progeny, the breeds were estimated to represent greater than 99% of the germplasm used in the US beef cattle industry, contain more than 187 unshared haploid genomes, and allow a 95% probability of detecting any allele with a frequency greater than 0.016 ^21^.

The second panel of 1142 cattle consisted of samples from male and female registered purebred cattle with diverse pedigrees from 46 breeds. Samples were from semen, blood, or hair follicles, depending on gender and availability ^22^. Where possible, animals within breed were chosen so they did not share parents or grandparents, and none were closely related to the 96 sires in the MBCDPv2.9. The breeds used in the second panel were: Angus (n = 24), Ankole-Watusi (n = 20), Ayrshire (n = 24), Beefmaster (n = 24), Belgian Blue (n = 24), Blonde d'Aquitaine (n = 24), Brahman (n = 23), Brahmousin (n = 24), Brangus (n = 24), Braunvieh (n = 24), Brown Swiss (n = 26), Charolais (n = 24), Chianina (n = 24), Corriente (n = 24), Devon (n = 23), Dexter (n = 22), Gelbvieh (n = 23), Guernsey (n = 23), Hereford (n = 24), Highland (n = 24), Holstein (n = 81), Indu-Brazil (n = 25), Jersey (n = 29), Limousin (n = 24), Maine-Anjou (n = 24), Marchigiana (n = 24), Mini-Hereford (n = 24), Mini-Zebu (n = 24), Montbeliard (n = 24), Murray Grey (n = 20), Nelore (n = 24), Piedmontese (n = 25), Pinzgauer (n = 23), Red Angus (n = 23), Red Poll (n = 24), Romagnola (n = 24), Salers (n = 24), Santa Gertrudis (n = 24), Senepol (n = 23), Shorthorn (n = 23), Simmental (n = 23), Tarentaise (n = 24), Texas Longhorn (n = 23), Texas Longhorn, Cattlemen’s Texas Longhorn Registry (CTLR, n = 19), Tuli (n = 23), and Wagyu (n = 22).

### DNA sequencing and single nucleotide polymorphism (SNP) genotyping

Unless otherwise indicated, reagents were molecular-biology grade. DNA from whole blood samples was extracted by use of a solid-phase system incorporating either spin-columns or 96-well microtitration plates according to the manufacturer's instructions (Qiagen Inc., Germantown, MD, USA). DNA from liver, muscle, skin, or hair samples was extracted by standard procedures ^22^. Briefly, minced tissue (35 mg) or hair follicles (100 trimmed bulbs) were suspended in 2.5 mL of a lysis solution containing 10 mM TrisCl, 400 mM NaCl, 2 mM EDTA, 1% wt/vol sodium dodecyl sulfate, RNase A (250 ug/ml; Sigma-Aldrich, St. Louis, MO, USA), pH 8.0. The solution was incubated at 37°C with gentle agitation. After 1 hour, 1 mg proteinase K was added (Sigma-Aldrich) and the solution was incubated overnight at 37°C with continued agitation. The solution was transferred to 15 ml tube containing 3 ml of a phase-separation gel (high-vacuum grease, Dow Corning Corporation, Midland, MI, USA) and extracted twice with 1 vol of phenol:chloroform:isoamyl alcohol (25:24:1), and once with 1 vol of chloroform before precipitation with 2 vol of 100% ethanol. The precipitated DNA was washed once in 70% ethanol, briefly air dried, and dissolved in a solution of 10 mM TrisCl, 1 mM EDTA (TE, pH 8.0).

DNA from commercial bull semen was extracted similarly, with slight modification ^23^. Briefly, three 0.5 ml straws of commercial semen from a single animal were pooled, and the cells were collected by centrifugation for 5 min at 1000 x *g*. The cell pellet was washed three times in 1 ml of a wash solution (TE with 100 mM NaCl, TNE) and suspended in 1 ml of the same solution with 1% wt/vol sodium dodecyl sulfate, 1 mg proteinase K (Sigma-Aldrich), and 40 mM dithiothreitol (DTT). This 1 ml lysis solution was incubated overnight at 37°C, transferred to a 15 ml tube containing 1.5 ml of TNE with 40 mM DTT, and 3 ml of a phase-separation gel, extracted twice with 1 vol of phenol:chloroform:isoamyl alcohol (25:24:1), and once with 1 vol of chloroform before precipitation with 0.1 vol of 3 M sodium acetate (pH 5.2) and 2 vol of 100% ethanol. The precipitated DNA was washed once in 70% ethanol, briefly air dried, and dissolved in a solution TE.

PCR-amplified fragments of genomic DNA from *ITGB2* exons 2, 3 and 5, encoding the CD18 signal peptide sequence and the region containing the known variant causing BLAD, were produced for Sanger sequencing in 96 or 384-well plates. A standard 25 µl amplification reaction contained 2.5 µl of genomic DNA in TE (10 ng/µl), 12.5 µl of a concentrated PCR cocktail (Maxima Hot Start, Thermo Fisher Scientific, Waltham, MA, USA), 1.25 µl each of an oligonucleotide primer stock solution (100 uM, in TE), 1 µl dimethyl sulfoxide (Sigma-Aldrich), and 6.5 µl water. The sense and antisense primer sequences for exons 2 and 3 were 5'-AGG-GAG-ACT-GAC-CTG-TGT-G-3' and 5'-CTG-GGA-AGC-AGA-GTG-ATA-GT-3', respectively (USMARC primer no. 89878 and 89880). The sense and antisense primer sequences for exon 5 were 5'-AGA-GAG-ATC-CAG-GTA-GAA-CTG-3' and 5'-GTG-CAG-AGG-TGC-AGA-GGT-G-3', respectively (USMARC primer no. 89887 and 89889). The final concentration of each primer was of 5 uM (Integrated DNA Technologies, Inc., Coralville, IA, USA). PCR was performed with either the PTC 200, the PTC 220 Dyad, or the PTC 225 Tetrad thermal cycler chassis (MJ Research, Watertown, MA, USA). Reactions were denatured at 94°C for 15 min, subjected to 45 cycles of denaturation at 94°C for 20 s, annealed at 58°C for 30 s, and extended at 72°C for 1 min. After cycling the final products were extended at 72°C for 3 min before storage at 4°C. A 5 µl portion of each amplified product was analyzed by agarose gel electrophoresis (0.8%) in buffer containing 90 mM Tris-borate (pH 8.0), 2 mM ethylenediamine tetraacetic acid, and 0.1 µg/ml ethidium bromide. A 6 µl portion of each amplified product was treated with Exonuclease I (1.4 U, New England Biolabs Inc., Ipswich, MA, USA) at 37°C for 1 hr in a 13 µl reaction volume to digest single-stranded primer oligonucleotides. The Exonuclease I was inactivated with a 65°C incubation for 20 min and the DNA was precipitated with two volumes of 100% ethanol. The plates were centrifuged at 1800 x g for 30 min, decanted, and air dried.

Sequencing reactions were accomplished by dissolving air-dried DNA pellets in 5 µl of sequencing reaction cocktail containing 0.25 µl of dye terminators (DYEnamic ET Dye terminators, Amersham Biosciences, Piscataway, NJ, USA), 1.75 µl dye terminator dilution buffer (Amersham Biosciences), 2 µl oligonucleotide primer (1.6 µM stock solution in water), and 1 µl water according to the manufacturer’s instructions. The final oligonucleotide concentration was 640 nM. Reactions were denatured at 96°C for 30 s, subjected to 26 cycles of denaturation at 96°C for 10 s, annealed at 50°C for 5 s, and extended at 60°C for 4 min. After cycling the final products were stored at 4°C until the DNA was precipitated with 22 µl of 70% isopropyl alcohol. The plates were centrifuged, at 1800 x g for 30 min, decanted, and the samples were washed with 22 µl of 70% ethanol. The plates were centrifuged again, decanted, air dried, sealed with foil, and stored at -20°C until use. Sequencing reactions were resolved by capillary electrophoresis as described by the manufacturer (3730xl DNA Analyzer, Applied Biosystems, Foster City, CA, USA). Animal sequences from both strands were analyzed with polyphred software version 6.18 ^24^ in conjunction with the phred/phrap/consed software version 29 ^25– 27^.

### Whole genome sequencing of BL3 cells

Whole genome sequencing of BL3 cells (a bovine lymphoma cell line; kindly provided by Dr. Subramaniam Srikumaran) was accomplished with methods as described elsewhere ^20^. Briefly, genomic DNA was used to make a 500 bp paired-end library and sequenced with a massively parallel sequencing machine and high-output kits (NextSeq500, two by 150 paired-end reads, Illumina, San Diego, CA, USA) until a minimum of 40 GB of data with greater than Q20 quality, was collected. After sequencing, the raw reads were filtered to remove adaptor sequences, contaminating dimer sequences, and low-quality reads. The DNA sequence alignment process was similar to that previously reported ^20^. FASTQ files were aggregated for each sample and DNA sequences were aligned individually to the bovine reference assembly UMD3.1 ^28^ with the Burrows-Wheeler aligner (BWA) aln algorithm version 0.7.12 ^29^, then merged and collated with bwa sampe. The resulting sequence alignment map (SAM) files were converted to binary alignment map (BAM) files, and subsequently sorted via SAMtools version 0.1.18 ^30^. Potential PCR duplicates were marked in the BAM files using the Genome Analysis Toolkit (GATK) version 1.5-32-g2761da9 ^31^. Regions in the mapped dataset that would benefit from realignment due to small indels were identified with the GATK module RealignerTargetCreator, and realigned using the module IndelRealigner. The BAM files produced at each of these steps were indexed using SAMtools. The resulting indexed BAM files were made available via the USMARC WGS browser and the raw reads for the BL3 cell line were deposited at NCBI BioProject PRJNA325058, BioSample number SAMN05217649. Mapped datasets for each sample were individually genotyped with the GATK UnifiedGenotyper with arguments “--alleles” set to the VCF file ( Extended Data File S1) ^32^, “--genotyping_mode” set to “GENOTYPE_GIVEN_ALLELES”, and “--output_mode” set to “EMIT_ALL_SITES”. Lastly, some SNP variants were identified manually by inspecting the sequence with IGV software version 2.1.28 ^33, 34^ (described in the Methods section entitled ‘Identifying protein variants encoded by *ITGB2*’). In these cases, read depth, allele count, allele position in the read, and quality score were considered when the manual genotype determination was made.

### Identifying predicted protein variants encoded by bovine *ITGB2*

Aligned WGS data from 96 sires of MBCDPv2.9 were visually analyzed in the *ITGB2* coding region to identify potential CD18 protein variants. Viewing the aligned sequences and detecting variants was accomplished with the IGV software and a browser developed for this purpose. Briefly, public internet sites at the USDA, ARS, USMARC were used in combination with open source software installed on a laptop computer and recorded manually in a spreadsheet as previously described ^20^. A Java Runtime Environment (Oracle Corporation, Redwood Shores, CA, USA) was first installed on the computer. When links to the data were selected by the user, IGV software ^33, 34^ was loaded from a third-party site (University of Louisville, Louisville, KY, USA) and aligned DNA sequence reads were displayed in the context of the bovine UMD3.1 reference genome assembly. For viewing *ITGB2* gene variants, WGS from a set of eight animals of different breeds was loaded, and the IGV browser was directed to the appropriate genome region by entering “ *ITGB2*” in the search field. The IGV zoom function was used to view the first exon at nucleotide resolution with the [show translation] option selected in IGV. An example of the alignment view for *ITGB2* codon 27 with eight animals is shown in Extended data, Figure S1 ^35^.

The exon sequences were visually scanned for polymorphisms predicted to alter amino acid sequences, including missense, nonsense, frameshift, splice site, and insertion/deletion mutations. An *in silico* analysis of other potential splice-affecting variants was not performed as there are no consensus guidelines on the selection of programs or protocols to interpret the predicted results in cattle. Once identified, the variant nucleotide position was viewed and recorded for all 96 animals. The codons affected by SNP alleles were translated into their corresponding amino acids with IGV, codon tables, and knowledge of the CD18 protein sequence (NP_786975). Haplotype phases of predicted polypeptide variants were unambiguously assigned with homozygous individuals, and those with only one variant amino acid. A maximum parsimony phylogenetic tree was manually constructed from the unambiguously phased protein variants and used to infer phases in the remaining variants with maximum parsimony assumptions.

### MALDI-TOF MS genotyping of 14 *ITGB2* missense mutations

A single multiplex assay was designed for the 14 *ITGB2* missense SNPs with software provided by the manufacturer (Agena Biosciences, San Diego, CA, USA). The oligonucleotide sequences and assay conditions are provided in Table S1. After design and validation with bovine control DNAs for each SNP, the DNA from the 96 bulls in the MBCDPv2.9 diversity panel were tested in a blinded experiment. Assay design and genotyping was performed at GeneSeek (Lincoln, NE, USA) with the MassARRAY platform and iPLEX Gold chemistry according to the manufacturer’s instructions (Agena Biosciences).

### Lkt preparation, gel electophoresis, and protein immunoblotting

*M. haemolytica* strains for toxin production were isolated from cattle with severe fibrinous pleuropneumonia in feedlot environments and had complete closed whole genome sequence assemblies available at NCBI. *M. haemolytica* strain 89010807 N serotype A1 ( *lktA+*) has been widely used for Lkt production for *in vitro* cytotoxicity assays and has the added advantage of being the parent strain of an isogenic leukotoxin deletion mutant ( *lktA*-) ^36, 37^. A second strain, *M. haemolytica* strain USDA-ARS-USMARC-183 serotype A1, was isolated from an animal that was part of a high-mortality respiratory disease outbreak in a Kansas feedlot in 1991 and represents the first strain with a complete closed genome assembly ^38^; however, it had not previously been used in *in vitro* assays.

Isolates were maintained on Brain Heart Infusion (BHI) agar (Sigma-Aldrich) frozen stocks were kept in BHI broth with 20% glycerol at -80°C. RPMI 1640 medium (without Phenol Red and L-glutamine, Sigma-Aldrich) and semi-defined medium 2 (SDM2) were used for batch culture production of Lkt. SDM2 is an amino acid-limited culture medium supplemented with cysteine, glutamine, ferric iron, and manganese and was previously shown to greatly improve Lkt production in aerobic batch culture ^39^. For Lkt production, a single, 24-hour colony isolate from BHI agar was inoculated into 5 ml BHI broth in a 10 ml culture tube and incubated overnight at 37°C in 5% CO _2_ without shaking. The following morning, 1 ml of BHI liquid culture was inoculated into 100 ml of fresh culture medium in a 300 ml Delong-style Erlenmeyer flask with baffles (Corning, Inc., Corning, NY, USA) and incubated at 37°C, 250 rpm, in 5% CO _2_. At intervals, 14 ml samples were removed and centrifuged at 13,100 x g for 10 min at 4°C. The clarified supernatant was decanted, flash frozen in liquid nitrogen and stored at -80°C until use.

Lkt and other proteins secreted into the growth media by *M. haemolytica* were analyzed by SDS-PAGE. Clarified supernatants were precipitated with one volume of acetone on ice for 30 min, followed by centrifugation at 20,800 x g for 5 minutes at room temperature, and air dried 30 min. Sedimented proteins were dissolved in a commercial sample buffer with lithium dodecyl sulfate and dithiothreitol and used per the manufacture instructions (Thermo Fisher Scientific). Samples were heated to 70°C for 10 minutes and loaded on 4–12% precast polyacrylamide Bis-Tris gels (Thermo Fisher Scientific) at run 160 volts for approximately 35 min in 2-[N-morpholino]ethanesulfonic acid (MES) SDS running buffer (Thermo Fisher Scientific). Proteins sorted by SDS PAGE were stained with coomassie-dye reagent (GelCode Blue, Thermo Fisher Scientific) and destained in water. Prestained protein standards (Novex Sharp, Thermo Fisher Scientific) were used to estimate molecular weights of *M. haemolytica* proteins from clarified supernatants. ImageJ software (version 1.52A) was used to estimate relative proportions of protein bands on coomassie-stained page gel ^40^.

For protein immunoblots (western blots), proteins from SDS-PAGE gels were electrophoretically transferred to 0.2 µm polyvinylidene difluoride membranes (PVDF, Invitrolon, Thermo Fisher Scientific) with a Mini Blot Module (Thermo Fisher Scientific) per the manufacturer's instructions. PVDF membranes were wetted in 100% methanol prior to equilibrating in transfer buffer (Bolt transfer buffer, Thermo Fisher Scientific) and assembling in the blotting apparatus. Proteins were transferred to membranes for 60 min at a constant voltage of 20 V. Blots were removed from the apparatus and incubated in a blocking reagent (StartingBlock(PBS), Thermo Fisher Scientific), for 60 min at room temperature with gentle agitation. This solution was replaced with a fresh blocking reagent that had a rabbit polyclonal Ltk antibody at a concentration of 1 µg/ml ( *M. haemolytica* Lkt Antibody, catalog number LS-C369014, LifeSpan, BioSciences, Inc, Seattle, WA, USA) and incubated as above for 60 min. The primary Lkt antibody was washed three times in for 10 min each in 25 mM Tris, 0.15 M NaCl, 0.05% Tween-20, pH 7.5 (TBS Tween-20, Thermo Fisher Scientific). After washing, a goat anti-rabbit IgG antibody conjugated to horseradish peroxidase (HRP) (catalog number ab97040, Abcam, Cambridge, MA, USA) was added at a concentration of 1 µg/ml in blocking reagent and incubated for 60 min at room temperature with gentle agitation. This secondary anti-rabbit antibody was washed three times for 10 min each in TBS Tween-20 prior to detection with chemiluminescent substrate (Pierce ECL Western Blotting substrate, Thermo Fisher Scientific). The immunoblot was incubated in chemiluminescent substrate for 1 min and imaged for approximately 5 min (ChemiDoc, Bio-Rad Laboratories, Inc. Hercules, CA, USA).

### Cell culture and synthetic peptides

BL3 cells were propagated in RPMI 1640 medium (Gibco, Thermo Fisher Scientific) supplemented with 10% fetal bovine serum (Atlas Biologicals, Fort Collins, CO, USA), ****1x antibiotic/antimycotic (Gibco, Thermo Fisher Scientific) and 2 mM L-glutamine (Gibco, Thermo Fisher Scientific). Custom bovine CD18 signal peptides ( Figure 4– Figure 7) were commercially synthesized, (Thermo Fisher Scientific) purified by preparative high-performance liquid chromatography, and lyophilized. Peptides were dissolved in dimethysulfoxide at a concentration of 10 mg/ml, aliquoted, and stored at −20°C.

### PBMC and PMN cell isolation

Primary cells were collected from two mixed breed animals (kept as part of the USMARC cattle population) that were each homozygous for the most common *ITGB2* haplotype (variant “1”). For isolation of primary bovine cells, 50 ml of blood were collected by jugular puncture using 16-guage needles into syringes containing EDTA as an anticoagulant. PMN were isolated using a standard hypotonic lysis procedure. Briefly, blood was spun for 25 min at 1000 x g at 4°C. Plasma and buffy coat layers were removed and discarded. Sterile water was added to the red blood cell (RBC) layer to lyse RBC followed by addition of 10X PBS to restore tonicity. PMN were isolated by centrifugation for 10 min at 250 x g at 4°C. The PMN cell pellet was washed three times with 1x PBS and the final cell pellet was resuspended in RPMI 1640 medium.

Peripheral blood mononuclear cells (PBMC) were isolated essentially as described ^41^. Briefly, PBMC were isolated over Ficoll-Paque Plus (GE Healthcare Bio-Sciences AB, Uppsala, Sweden), as per the manufacturer’s instructions, with modification. Briefly, 15 ml of whole blood mixed 1:1 with PBS was underlayed beneath 14 ml of the density gradient in a 50 ml conical tube. The tubes were then centrifuged for 45 min at 900 x g at room temp and with no brake. The PBMC layer was carefully removed and brought up to 45 ml in PBS in a new 50 ml conical tube followed by centrifugation for 15 min at 400 x g at 4°C with high brake. Erythrocytes were removed using RBC lysing buffer (Sigma-Aldrich). The PBMC pellet was further washed three times with 1x PBS and the final pellet was resuspended in RPMI 1640 medium.

### MTT dye-reduction cytotoxicity assay

The ability of different CD18 signal peptide variants to bind Lkt and inhibit Lkt-induced cytolysis was measured with the MTT dye-reduction cytotoxicity assay ^10^. The BL3 cell line was selected because it is the most well-studied, readily available, immortalized cell line susceptible to Ltk-induced cytolysis. CD18 signal peptides were tested at concentrations ranging from 50 μM to 0.195 μM. CD18 signal peptides were diluted using serial 2-fold dilutions in 96-well round bottom plates containing 50 µl/well of Lkt at a 50% toxicity end point titer in colorless RPMI 1640 medium without phenol red (Sigma-Aldrich). Synthetic signal peptides and Lkt preparations were pre-incubated for 1 hr on ice prior to the addition of 5 × 10 ^5^ cells/wells. These cells were added as a 50 ul suspension with a density of 1 × 10 ^7^ cells/ml in colorless RPMI. Cells were incubated with synthetic signal peptides and Lkt for 1 hr at 37°C in 5% CO _2_ and subsequently centrifuged at 600 x g for 7 min at 4°C and the supernatant was removed and discarded. Cells were resuspended in 100 µl of colorless RPMI and 20 µl 0.5% MTT (3-(4,5-dimethylthiazol-2-yl)-2,5-diphenyl-2 *H*-tetrazolium bromide; Sigma-Aldrich) and incubated for 20 min at 37°C in 5% CO _2,_ centrifuged as before, and the supernatant was removed and discarded. The purple formazan precipitate was then dissolved in 100 µl of acid isopropanol (0.04N HCl in isopropanol). Following a 5 min incubation, cellular debris was pelleted and the supernatant was transferred to a new 96-well plate. The optical density (OD) of the samples was measured at 570 nm and 690 nm. The background measurement at 690 nm was then subtracted from the 570 nm measurement to give the background adjusted OD. The percent cytotoxicity was calculated as follows: (1-(OD of toxin treated cells/OD of cells without toxin)) x 100. The percent inhibition of cytotoxicity in the presence of synthetic CD18 peptides was calculated as follows: ((percent cytotoxicity in the absence of peptide – percent cytotoxicity in the presence of peptide)/percent cytotoxicity in the absence of peptide) x 100. Given normal variability in the assay, values were occasionally obtained outside the range of 0 to 100% inhibition. Negative values were replaced with 0 and values greater than 100 were replaced with 100 for graphical presentation.

Fresh bovine PBMC or PMN were used for comparison to results obtained using immortalized BL3 cells. Cells were suspended in 50 µl colorless RPMI medium and 10 µl Biolog Redox Dye MB, containing 500 μM water-soluble tetrazolium (Biolog, Hayward, CA, USA). This dye was used because it was found to be more sensitive to changes in cellular respiration when assaying primary cells (data not shown). Cells were incubated for 3 hr at 37°C in 5% CO _2_, centrifuged as before, and the supernatant was transferred to a clean 96 well plate prior to the OD being measured at 590 nm and 750 nm. Calculations for percent cytotoxicity and percent inhibition were calculated using background adjusted OD values as described above.

### Statistical analyses of IC _50_ values

The half maximal inhibitory concentration (IC50) was estimated for each signal peptide variant to compare the concentration of peptide needed to block 50% of Lkt-induced cytotoxicity of susceptible bovine cells. Univariate nonlinear regression (NLIN procedure, SAS 9.4, SAS Institute Inc., Cary, NC, USA) was used to fit data from three replicates to the following equation: Y _ijk_ = T + (B - T)/(1 + (C _j_/IC50 _i_) ^S^_i_) + e _ijk_, where Y _ijk_ is the measured Lkt inhibition for the k ^th^ replicate of the i ^th^ peptide at the j ^th^ concentration (C _j_) of peptide. T is the estimated maximum, B is the estimated minimum of the fitted nonlinear relationship between peptide concentration and Lkt inhibition. S _i_ is the slope of the relationship at IC50 _i_, the estimated concentration that the i ^th^ peptide inhibits Lkt-induced cytotoxicity by 50%. e _ijk_ is random variation. Standard errors of IC50 _i_ were used for comparisons of estimates.

## Results

### Identifying *ITGB2* polymorphisms affecting the predicted amino acid sequence of CD18

**Figure 1.**
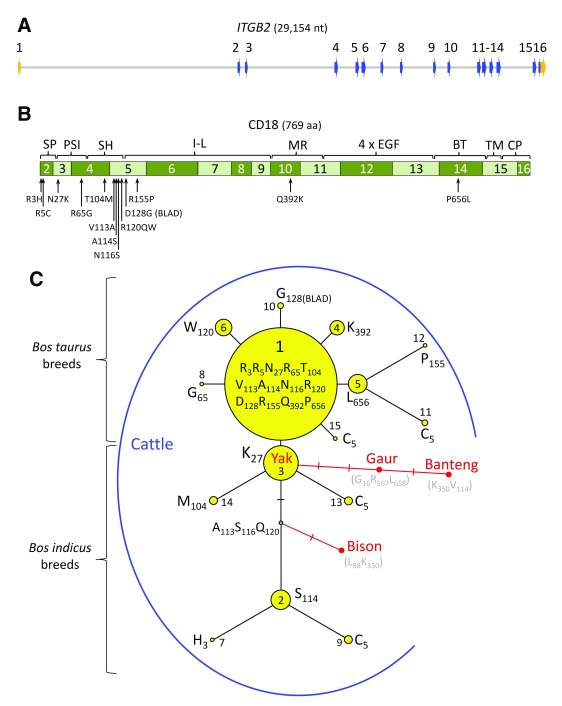
Physical maps of *ITGB2*, CD18, and rooted maximum parsimony phylogenetic tree of CD18 protein variants in cattle. ( **A**) Genomic DNA map of *ITGB2*: blue arrows, coding regions of exons; orange arrows, untranslated regions of exons; grey horizontal lines, intron regions. ( **B**) Map of CD18 domains in relationship to missense mutations found in cattle. ( **C**) Rooted maximum parsimony phylogenetic tree of CD18 protein variants from 1142 cattle from 46 breeds with connections to closely-related species. The most frequent CD18 isoform (“variant 1”) was used as the reference amino acid sequence for the trees. For “variants 1” through “14”, each node in the tree represents a different isoform of CD18 that varies by one amino acid compared to adjacent nodes. The hash marks represent nodes that were inferred but not observed. The areas of the circles are proportional to the overall variant frequency in the cattle tested. Yak CD18 was identical to bovine CD18 “variant 3” and denoted as the most likely root of the phylogenetic tree based on the relationship of CD18 sequences in yak, gaur, banteng, and bison. SP, signal peptide; PSI, plexin-semaphorin-integrin domain; SH, spacer-hybrid domain; I-L, I-like domain; MR, mid-region hybrid domain; EGF, epidermal growth factor-like domain; BT, beta tail domain; TM, transmembrane domain; CP, cytoplasmic domain.

Bovine *ITGB2* consists of 16 exons spanning 29.1 kb of genomic DNA and encodes a 769 amino acid protein with multiple functional domains ( Figure 1A,B). Using software to view the aligned genome sequences from 96 diverse bulls, 13 codons with 14 missense variants were identified ( Extended Data Table S2) ^42^. There were no frameshifts, splice sites, or indel polymorphisms observed that would affect the predicted amino acid sequence. Two *ITGB2* regions of interest were further selected for additional Sanger sequencing in 1142 purebred cattle from 46 breeds: the signal peptide region (exon 2), and the region containing the D128G variant causing BLAD (exon 5). DNA sequence analyses revealed no additional polymorphisms that were predicted to alter the polypeptide sequence, except the previously described D128G variant in Holstein cattle (data not shown). The genotypes were independently verified with a single, multiplexed, MALDI-TOF MS assay for 14 SNPs in the 96 diverse bulls, and in 1142 of 1168 cattle from 46 breeds ( Extended Data Table S3) ^43^. Thus, in total, there were 14 missense variants identified in 13 codons ( Table 1).

**Table 1.** DNA sequence information for 14 bovine *ITGB2* missense SNP variants in cattle.

						MAF ^e^		
Codon variant ^a^	Chr 1 position (UMD3.1)	Exon	CD18 domain ^b^	Consensus codon sequence ^c^	Codon alleles ^d^	Ref. panel	Ext. panel	Flanking reference genomic sequence
R3H	145121452	2	SP	c **R**c	c **G**c = R c **A**c = H	0.010	0.0004	ctggggtctgaagctatgtcactgccccttccctcctcaggacatgctgc **[R]**ccagcgcccccagctgctgctcctagcgggcctgcttgccctccagtccg
R5C	145121447	2	SP	**Y**gc	**C**gc = R **T**gc = C	0.005	0.0123	gtctgaagctatgtcactgccccttccctcctcaggacatgctgcgccag **[Y]**gcccccagctgctgctcctagcgggcctgcttgccctccagtccggtgag
N27K	145121043	3	PSI	aa **S**	aa **C** = N aa **G** = K	0.115	0.1187	ggctcaccagcccgtgtctctctcccagtcctgtcccaggagtgcaccaa **[S]**tacaaggtcagcacctgccgggactgcatcgagtcgggccccggctgcgc
R65G	145116134	4	PSI	**S**ga	**C**ga = R **G**ga = G	0.005	0.0004	cccagaacttcacagggcaaggggagcccgactccattcgctgtgacaca **[S]**gagcggagctgctgtcaaagggctgcccagctgatgacatcatggaaccc
T104M	145116016	4	SH	a **Y**g	a **C**g = T a **T**g = M	0.010	0.0035	ccgggacagccaggcgggcagtcggaagcagctgtccccacaggaagtga **[Y]**gctctacctgagaccaggtaggcttggctggctaggggtgggccggccct
V113A	145115008	5	SH	g **Y**t	g **T**t = V g **C**t = A	0.057	0.0299	gaccaggtggtacaccctgactctctcccaaatcctggcaggtcaggcag **[Y]**tgcgttcaatgtgaccttccggagggccaagggctaccccatcgacctgt
A114S	145115006	5	SH	**K**cg	**G**cg = A **T**cg = S	0.057	0.0298	ccaggtggtacaccctgactctctcccaaatcctggcaggtcaggcagtt **[K]**cgttcaatgtgaccttccggagggccaagggctaccccatcgacctgtac
N116S	145114999	5	SH	a **R**t	a **A**c = N a **G**c = S	0.057	0.0290	gtacaccctgactctctcccaaatcctggcaggtcaggcagttgcgttca **[R]**tgtgaccttccggagggccaagggctaccccatcgacctgtactacctga
R120W	145114988	5	SH	**Y**gg	**C**gg = R **T**gg = W	0.021	0.0171	ctctctcccaaatcctggcaggtcaggcagttgcgttcaatgtgaccttc **[Y]**ggagggccaagggctaccccatcgacctgtactacctgatggacctctcc
R120Q	145114987	5	SH	c **R**g	c **G**g = R c **A**g = Q	0.057	0.0317	tctctcccaaatcctggcaggtcaggcagttgcgttcaatgtgaccttcc **[R]**gagggccaagggctaccccatcgacctgtactacctgatggacctctcct
D128G ^h^	145114963	5	IL	g **R**c	g **A**c = D g **G**c = G	- ^g^	0.0031	ggcagttgcgttcaatgtgaccttccggagggccaagggctaccccatcg **[R]**cctgtactacctgatggacctctcctactccatggtggatgacctcgtca
R155P	145114882	5	SH	c **S**g	c **G**g = R c **C**g = P	0.005	0.0004	catggtggatgacctcgtcaacgtcaagaagctggggggtgacctgctcc **[S]**ggccctcaatggcatcaccgagtcgggccgcattggtgaggcagctactc
Q392K	145109830	10	MR	**M**ag	**C**ag = Q **A**ag = K	0.021	0.0131	ctgacaccctgaaagtcacctacgactccttctgcagtaacgggaaatcg **[M]**aggtggaccagcccagaggggactgcgacggcgtccagatcaacgtcccg
P656L	145107135	14	BT	c **Y**g	c **C**g = P c **T**g = L	0.010	0.0270	cgccaagaactgcagcgcagcgtgcgggcagacgaagctgctgtccagcc **[Y]**ggtgcccggccgcaagtgcaaggagcgcgactccgagggctgctggatga

^a^All sequences presented are from the sense strand bovine *ITGB2* gene. However, in the UMD3.1 reference assembly, *ITGB2* oriented in the antisense direction. ^b^CD18 protein domain abbreviations: SP, signal peptide; PSI, plexin-semaphorin-integrin domain; SH, spacer-hybrid domain; IL, I-like domain; and BT, beta-tail domain. ^c^IUPAC/IUBMB ambiguity codes used for nucleotides: R= a/g, Y = c/t, M= a/c, K = g/t, S = c/g, W = a/t ^46^. ^d^The major allele is listed first. ^e^Minor allele frequency in the beef cattle diversity panel MBCDPv2.9 (n = 96). ^f^Minor allele frequency in the extended purebred cattle panel with 46 breeds (n = 1142). ^g^Allele not detected in the indicated group of cattle. ^h^Missense mutation associated with BLAD ^17^.

### Determining *ITGB2* haplotypes encoding different CD18 polypeptides

Identifying haplotypes that encode distinct combinations of missense variants on the CD18 polypeptide is important for evaluating their potential function. A total of 15 *ITGB2* haplotypes were identified that, when translated, were predicted to encode different CD18 proteins ( Table 2). These 15 predicted polypeptide sequences were placed in the context of a maximum parsimony phylogenetic tree ( Figure 1C). Haplotypes encoding CD18 protein variants “1 to 7”, “9”, “13”, and “14” were confirmed by their presence in homozygous animals. Haplotypes encoding CD18 protein variants “8”, “10”, and “15” were unambiguously confirmed in animals with only one heterozygous site. However, haplotype phase was ambiguous when the distance between two heterozygous sites exceeds the length of the DNA sequence read (150 bp in these WGS data sets). Thus, haplotypes for the remaining CD18 protein variants “11” and “12” were tentatively inferred from additional breed-level frequency information. For example, the inferred phase for variant “12” (P _155_L _656_) was only observed in two of 27 Braunvieh cattle that were each heterozygous for both missense variants. However, all 25 of the other Braunvieh cattle sequenced were homozygous for the variant “1” ( Table 3 and Extended Data Table S3) ^43^. Thus, it was reasonable to infer that P _155_ (exon 5), and L _656_ (exon 14) are present on one rare haplotype in two animals, rather than on two rare haplotypes in each animal. Similarly, the inferred phase for variant “11” (C _5_L _656_) was only observed in six of 22 Wagyu cattle and each were heterozygous at positions 5 and 656. Since the 22 Wagyu cattle have a variant “1” frequency of 0.8 it seems likely that C _5_ and L _656_ variants are present on the same chromosome (i.e. diplotype “1,11”) rather than split across two chromosomes (i.e., diplotype “5,15”). In spite of the potential for ambiguous haplotype phases with rare variants, the phylogenetic tree of predicted CD18 proteins provides a solid framework for further evaluation.

**Table 2.** Frequencies of predicted full-length CD18 protein variants in U.S. cattle.

		Protein variant frequency ^c^	
Protein variant code ^a^	Variant amino acids ^b^	Beef cattle diversity panel (n = 96)	Extended purebred cattle panel (n = 1142)
1	R3 R5 N27 R65 T104 V113 A114 N116 R120 D128 R155 Q392 P656	0.828	0.820
2	R3 R5 **K27** R65 T104 **A113 S114 S116 Q120** D128 R155 Q392 P656	0.042	0.025
3	R3 R5 **K27** R65 T104 V113 A114 N116 R120 D128 R155 Q392 P656	0.047	0.081
4	R3 R5 N27 R65 T104 V113 A114 N116 R120 D128 R155 **K392** P656	0.021	0.013
5	R3 R5 N27 R65 T104 V113 A114 N116 R120 D128 R155 Q392 **L656**	0.005	0.024
6	R3 R5 N27 R65 T104 V113 A114 N116 **W120** D128 R155 Q392 P656	0.021	0.017
7	**H3** R5 **K27** R65 T104 **A113 S114 S116 Q120** D128 R155 Q392 P656	0.010	- ^d^
8	R3 R5 N27 **G65** T104 V113 A114 N116 R120 D128 R155 Q392 P656	0.005	-
9	R3 **C5 K27** R65 T104 **A113 S114 S116 Q120** D128 R155 Q392 P656	0.005	0.004
10	R3 R5 N27 R65 T104 V113 A114 N116 R120 **G128** R155 Q392 P656	-	0.003
11	R3 **C5** N27 R65 T104 V113 A114 N116 R120 D128 R155 Q392 **L656**	-	0.003
12	R3 R5 N27 R65 T104 V113 A114 N116 R120 D128 **P155** Q392 **L656**	0.005	-
13	R3 **C5 K27** R65 T104 V113 A114 N116 R120 D128 R155 Q392 P656	-	0.005
14	R3 R5 **K27** R65 **M104** V113 A114 N116 R120 D128 R155 Q392 P656	0.010	0.004
15	R3 **C5** N27 R65 T104 V113 A114 N116 R120 D128 R155 Q392 P656	-	0.001

^a^CD18 protein variant allele definitions are shown in Figure 4C. ^b^The red bold residues are those differing from “variant 1”. ^c^The coefficient of determination for these frequencies (r ^2^) was 99.7. ^d^Allele not detected in the indicated group of cattle.

**Table 3.** Frequencies of predicted CD18 protein variants in 46 U.S. breeds.

			CD18 protein variant allele frequency ^a^														
Breed group	Animals typed (n=1142)	C5 ^b^	1	2	3	4	5	6	7	8	9 ^b^	10 ^c^	11 ^b^	12	13 ^b^	14	15 ^b^
Angus	24	-	0.96	-	-	0.04	-	-	-	-	-	-	-	-	-	-	-
Ankole-Watusi	20	-	0.25	-	0.75	-	-	-	-	-	-	-	-	-	-	-	-
Ayrshire	24	-	1.00	-	-	-	-	-	-	-	-	-	-	-	-	-	-
Beefmaster	24	-	0.88	0.08	0.02	-	-	-	-	-	-	-	-	-	-	0.02	-
Belgian Blue	24	-	0.90	-	-	0.04	0.06	-	-	-	-	-	-	-	-	-	-
Blonde d'Aquitaine	24	-	0.77	-	-	0.04	0.08	0.10	-	-	-	-	-	-	-	-	-
Brahman	23	-	0.11	0.27	0.58	-	0.02	-	0.02	-	-	-	-	-	-	-	-
Brahmousin	24	0.10	0.71	0.06	0.13	-	-	-	-	-	0.10	-	-	-	-	-	-
Brangus	24	-	0.55	0.09	0.21	0.15	-	-	-	-	-	-	-	-	-	-	-
Braunvieh	24	-	0.98	-	-	-	-	-	-	-	-	-	-	0.02	-	-	-
Brown Swiss	26	-	0.98	-	-	-	0.02	-	-	-	-	-	-	-	-	-	-
Charolais	24	0.02	0.79	-	0.06	-	-	0.13	-	-	-	-	-	-	0.02	-	-
Chianina	24	-	0.98	-	-	0.02	-	-	-	-	-	-	-	-	-	-	-
Corriente	24	-	1.00	-	-	-	-	-	-	-	-	-	-	-	-	-	-
Devon	23	-	0.87	-	-	0.04	0.09	-	-	-	-	-	-	-	-	-	-
Dexter	22	-	1.00	-	-	-	-	-	-	-	-	-	-	-	-	-	-
Gelbvieh	23	-	0.70	-	-	-	0.07	0.24	-	-	-	-	-	-	-	-	-
Guernsey	23	-	0.96	-	-	0.02	0.02	-	-	-	-	-	-	-	-	-	-
Hereford	24	-	0.98	-	-	-	-	0.02	-	-	-	-	-	-	-	-	-
Highland	24	-	1.00	-	-	-	-	-	-	-	-	-	-	-	-	-	-
Holstein	81	-	0.93	-	-	0.02	0.01	-	-	-	-	0.04	-	-	-	-	-
Indu-Brazil	25	0.09	-	0.32	0.55	-	0.02	-	-	-	0.06	-	-	-	0.02	0.02	-
Jersey	29	-	1.00	-	-	-	-	-	-	-	-	-	-	-	-	-	-
Limousin	24	-	0.90	-	-	0.10	-	-	-	-	-	-	-	-	-	-	-
Maine-Anjou	24	-	0.96	-	-	-	-	0.04	-	-	-	-	-	-	-	-	-
Marchigiana	24	-	1.00	-	-	-	-	-	-	-	-	-	-	-	-	-	-
Mini Hereford	24	-	1.00	-	-	-	-	-	-	-	-	-	-	-	-	-	-
Mini Zebu	24	-	0.33	0.15	0.48	-	-	-	-	-	-	-	-	-	-	0.04	-
Montbeliard	24	-	0.94	-	-	-	0.06	-	-	-	-	-	-	-	-	-	-
Murray Gray	20	-	0.78	-	-	-	0.23	-	-	-	-	-	-	-	-	-	-
Nelore	24	-	-	0.16	0.82	-	-	-	-	-	-	-	-	-	-	0.02	-
Piedmontese	25	-	0.92	-	-	-	0.08	-	-	-	-	-	-	-	-	-	-
Pinzgauer	23	-	0.89	-	-	-	0.11	-	-	-	-	-	-	-	-	-	-
Red Angus	24	-	1.00	-	-	-	-	-	-	-	-	-	-	-	-	-	-
Red Poll	23	-	0.98	-	-	-	-	0.02	-	-	-	-	-	-	-	-	-
Romagnola	24	-	1.00	-	-	-	-	-	-	-	-	-	-	-	-	-	-
Salers	24	-	0.77	-	-	-	-	0.21	-	0.02	-	-	-	-	-	-	-
Santa Gertrudis	24	0.02	0.63	0.02	0.25	-	0.02	-	-	-	0.02	-	-	-	-	0.06	-
Senepol	23	-	0.89	0.04	-	-	0.07	-	-	-	-	-	-	-	-	-	-
Shorthorn	23	-	0.96	-	-	-	0.04	-	-	-	-	-	-	-	-	-	-
Simmental	23	-	0.87	-	-	-	0.09	0.04	-	-	-	-	-	-	-	-	-
Tarentaise	24	-	0.94	-	-	0.02	0.02	0.02	-	-	-	-	-	-	-	-	-
Texas Longhorn	23	-	0.74	0.07	0.15	0.04	-	-	-	-	-	-	-	-	-	-	-
T. Longhorn, CTLR ^e^	19	-	0.95	-	0.05	-	-	-	-	-	-	-	-	-	-	-	-
Tuli	23	0.04	0.91	-	0.04	-	-	-	-	-	-	-	-	-	-	-	0.04
Wagyu	22	0.14	0.80	-	-	-	0.07	-	-	-	-	-	0.14	-	-	-	-

^a^CD18 protein variant allele definitions are shown in Table 2. ^b^The CD18 C5 missense variant appears in protein variants 9, 11, 13, and 15. ^c^The CD18 G128 missense variant causes BLAD in Holstein cattle. ^d^Allele not detected in the indicated group of cattle. ^e^Texas Longhorn, Cattlemen’s Texas Longhorn Registry.

### Evolutionary comparison of CD18 polypeptide sequences

Determining the most likely phylogenetic root of the CD18 tree is important for establishing the likely order of mutational events. Comparing cattle CD18 precursor protein variants to those from closely related species in the *Bos* genus indicated that variant “3” (K _27_) was the most likely root of the phylogenetic tree ( Figure 1C). Cattle are predicted to share a common ancestor with other species in the *Bos* genus approximately 5 million years ago. In addition, the K _27_ variant was associated with indicine cattle breeds, while the N _27_ variant was associated with taurine breeds. For example, the N _27_ frequency in 840 cattle from 33 taurine breeds was 0.998, while the K _27_ frequency in 70 Brahman, Indu Brazil, and Nelore cattle was 0.95. Thus, the structure of the rooted tree suggests that CD18 protein variants “1” and “3” are the ancestral polypeptide sequence of taurine and indicine breeds, respectively. The rooted tree also suggests that the distal nodes represent CD18 variants that have arisen sometime after the split between taurines and indicines, approximately 500,000 years ago.

**Figure 2.**
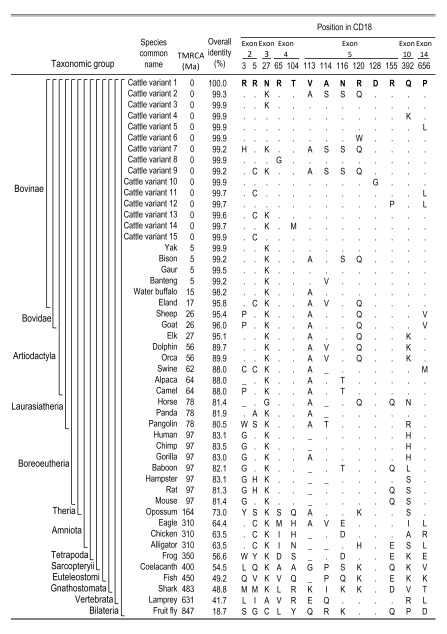
Evolutionary comparison of CD18 residues at their variant sites in U.S. cattle. Aligned and gapped protein sequences from a representative set of 56 bilateria species were compared ( Extended Data Table S4). At variant sites in cattle, the residues were summarized for a representative subset of 35 species. TMRCA, estimated time to most recent common ancestor in millions of years ^47^; letters, IUPAC/IUBMB codes for amino acids; dot, amino acid residues identical to those in cattle “variant 1”; dash, not enough sequence similarity for comparison or missing residue in that peptide region.

The conservation of amino acid residues throughout vertebrate species is a measure of their potential impact on protein function. Highly conserved residues are more likely to be indispensable for function and thus, variation at these positions is assumed to be deleterious. The 769 amino acid sequence of cattle CD18 is highly similar to those from yak, sheep, whale, and humans (99, 95, 90, and 83% identity, respectively). The cattle CD18 sequence is also remarkably similar to chicken, frog, fish, lamprey, and fruit fly (63, 57, 49, 42, and 19% identity, respectively), and has some polypeptide regions that are invariant throughout the Bilateria ( Extended Data Table S4) ^44^. The 13 variant amino acid positions in the bovine CD18 precursor protein were compared to those in 35 representative Bilateria species. The aspartate residue at position 128 (D _128_) is invariant throughout the Bilateria, except in cattle wherein G _128_ causes the complete loss of CD18 function and results in BLAD in homozygous individuals of the Holstein breed ( Figure 2). CD18 positions 65, 104, and 155 are also conserved and the variant allele in cattle is rare. Based only on the degree of residue conservation across species, the proposed predicted order of negative impact on CD18 function in cattle was: G _128_ > G _65_ = M _104_ > P _155_. The CD18 K _27_ indicine variant was conserved throughout the jawed vertebrates and thus, the major N _27_ taurine variant was not observed in any other species. However, based on its high frequency in most taurines, the N _27_ variant does not appear to be deleterious.

The conservation of arginine at positions 3 (R _3_) and 5 (R _5_) in CD18 signal peptides was of particular interest because this region binds bacterial Lkt in ruminant species, which includes the Bovids, Cervids, Giraffids, musk deer, chevrotains, and pronghorns. The R _3_ residue was conserved throughout the Bovinae, but not in sheep and goat, which have proline at that position ( Figure 2). The histidine residue at position 3 (H _3_) was rare in cattle, not observed in other ruminants, and on a distal node of the phylogenetic tree (CD18 polypeptide variant “7“, Figure 1C). Two animals from the Brahman breed group were identified with the H _3_ and one was homozygous, indicating that H _3_ is not a lethal recessive variant (H _3_,H _3_, Extended Data Table S2 ^45^, and R _3_,H _3_, Extended Data Table S3 ^43^). Unlike R _3_, the R _5_ variant was not conserved in Bovinae, since eland have cysteine at this site (C _5_). In addition, the C _5_ variant was present on four distinct putative CD18 polypeptide variants: “9“, “11”, “13”, and “15” ( Figure 1C), including both taurine- and indicine-influenced breeds. The presence of the C _5_ variant on multiple but infrequent haplotypes indicates recombination has occurred between this and other CD18 missense variants.

### Comparison of batch production methods of biologically-active Lkt with two reference strains of *M. haemolytica*

**Figure 3.**
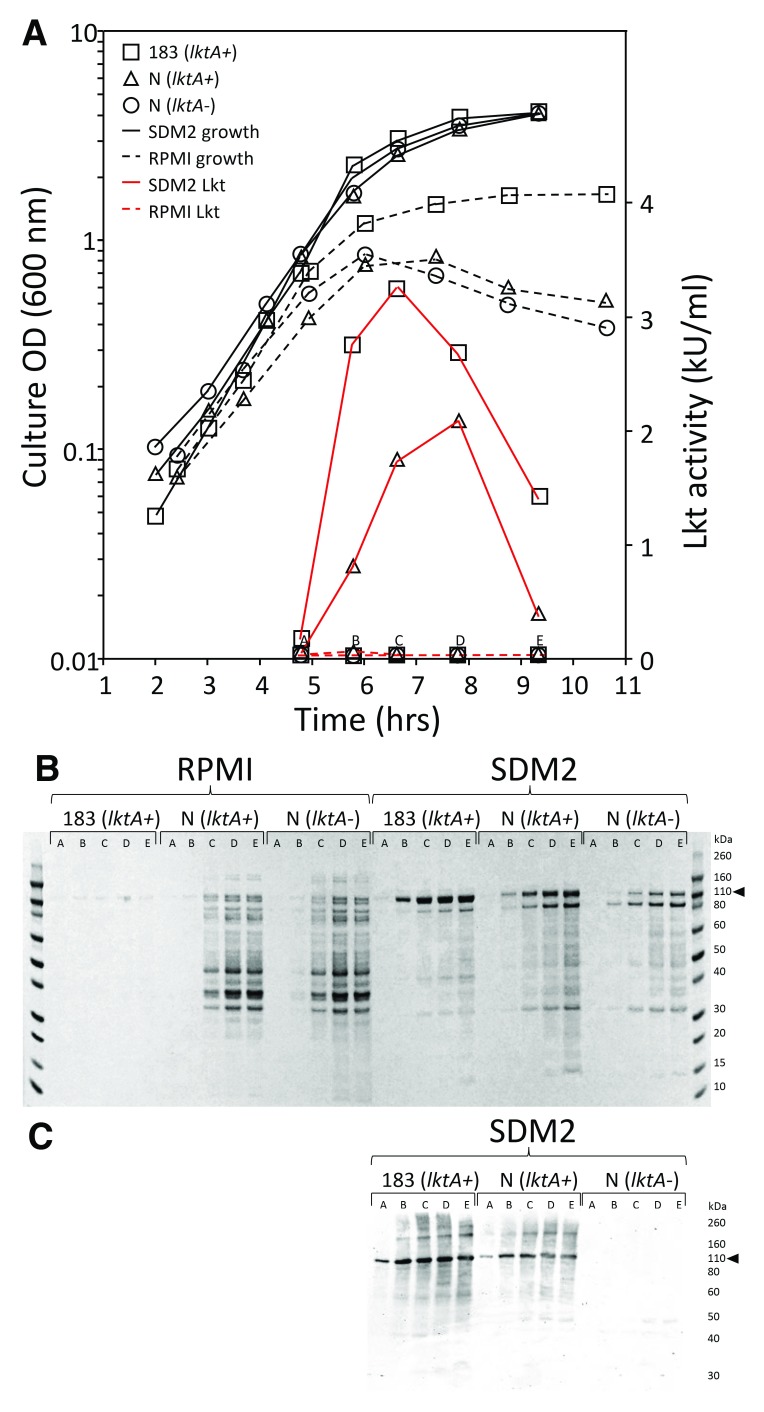
Comparison of *M. haemolytica* leukotoxin (Lkt) production in cell culture medium and a semi-defined bacterial culture broth. ( **A**) Growth curves of *M. haemolytica* strains (black lines) and the cytolytic activity of their secreted Lkt (red lines). The strain abbreviations: 183 ( *ltkA+*), USDA-ARS-USMARC-183 ( *ltkA+*); N ( *ltkA+*), 89010807N ( *lktA+*); and N ( *ltkA-*), 89010807N ( *lktA-*). Clarified culture supernatants were collected at time points indicated on the x-axis as A through E. ( **B**) Coomassie-stained SDS-PAGE of proteins from 50 µl each of clarified culture supernatants. ( **C**) Western blot of SDS-PAGE from the same clarified SDM2 culture supernatants that were used in panel ( **B**). The black arrow indicates the expected position of Lkt on SDS-PAGE.

The discovery of missense variants in the CD18 signal peptide provided the opportunity to test these variants using *in vitro* cell assays with bacterial Lkt. However, producing sufficient and consistent batches of biologically-active Lkt from reference bacterial strains was a challenge with traditional cell culture medium (RPMI). In an effort to overcome this barrier, Lkt production in RPMI was compared with that in a semi-defined bacterial culture medium (SDM2, Methods) with two wild-type *M. haemolytica* strains. The total biological activity of Lkt excreted into SDM2 culture was up to 80-fold greater at its peak than that in RPMI for a given reference strain of *M. haemolytica* (e.g. Strain 183, Figure 3A). In both media, Lkt activity was induced in late log phase as batch cultures made the transition to stationary phase. Although the biological activity quickly diminished as the culture progressed to stationary phase, the total Lkt protein measured by SDS PAGE continued to increase and the level was stable for hours in the culture supernatant ( Figure 3B). The Lkt of clarified SDM2 culture supernatants with the highest cytolytic activity (Strain 183, fraction C, Figure 3B) was estimated to be 90% pure based on gel densitometry imaging of coomassie-stained SDS-PAGE gels. Preparations of similar quality were used for *in vitro* assays. The cytolytic activity of these toxin preparations was stable at -80°C for more than 2 years.

**Figure 4.**
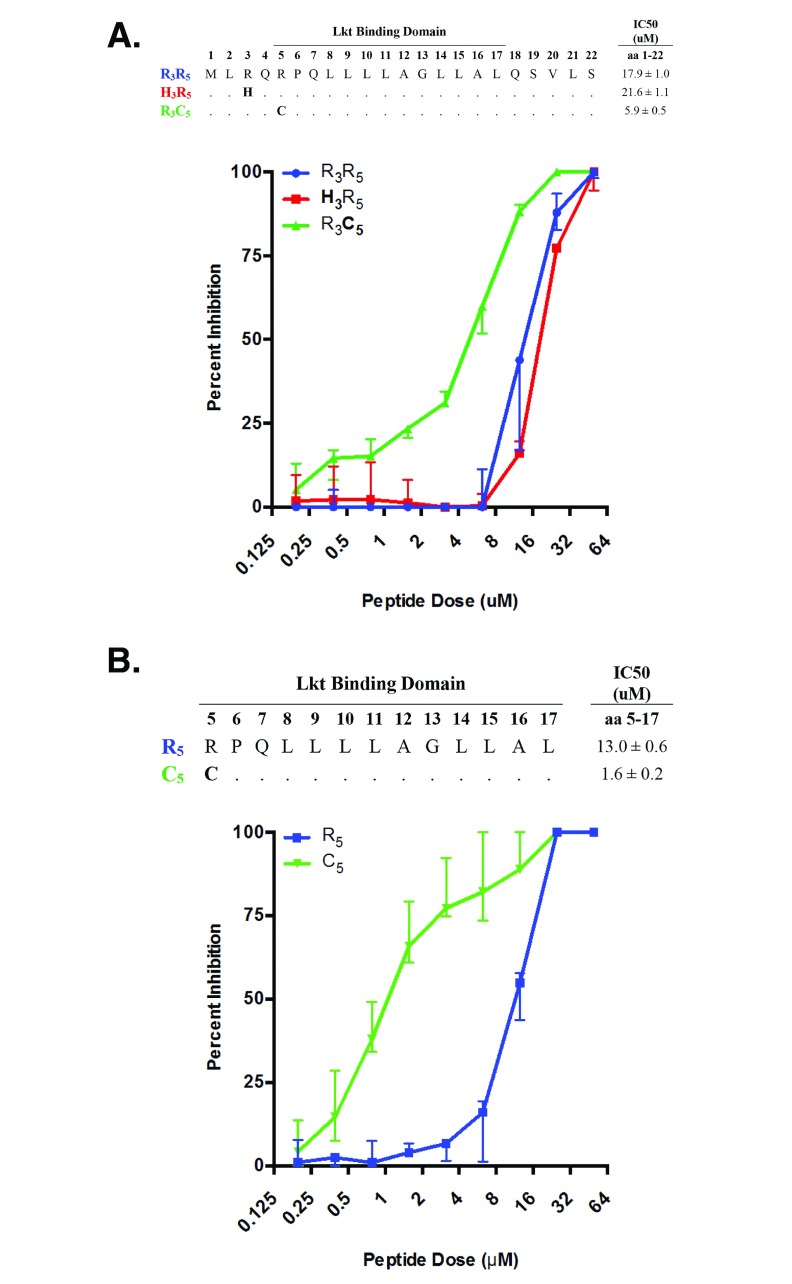
Cattle CD18 signal peptide variants differentially inhibit leukotoxin (Lkt)-induced cytolysis of bovine BL3 cells. CD18 signal peptide variants were tested for their ability to bind Lkt and inhibit Lkt-induced cytolysis of immortalized bovine BL3 cells using a MTT dye reduction assay. CD18 peptide variants representing amino acids 1 to 22 ( **A**) or 5 to 17 ( **B**) were tested using 2-fold serial dilutions at concentrations ranging from 50 μM to 0.195 μM. Half-maximal inhibitory concentration (IC50) for each peptide was determined using non-linear regression analyses. Data are expressed as the mean with standard deviation (n=3).

**Figure 5.**
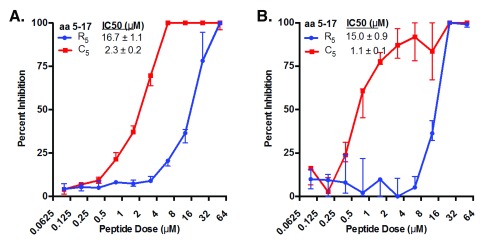
Cattle CD18 signal peptides inhibit leukotoxin (Lkt)-induced cytolysis of primary bovine cells. The 13-mer synthetic signal peptides (from Figure 4B) containing cattle CD18 residues 5 to 17 were tested for their ability to inhibit Lkt-induced cytolysis of primary bovine peripheral blood mononuclear cells (PBMC) ( **A**) and polymorphonuclear leukocytes (PMN) ( **B**). Donor animals were genotyped at the CD18 locus and were homozygous for CD18 variant “1”. Peptides were tested using 2-fold dilutions at concentrations ranging from 50 μM to 0.098 μM. The half-maximal inhibitory concentration (IC50) for each peptide was determined using non-linear regression analyses. Data are expressed as the mean with standard deviation (n = 3).

### CD18 signal peptide variant C _5_ has enhanced binding to bacterial Lkt

Analysis of cattle *ITGB2* haplotypes showed that three distinct polypeptide sequences are encoded in the 22-amino acid signal peptide region: the common variant with arginine at positions 3 and 5 (R _3_R _5_), a rare variant with histidine at position three (H _3_R _5_), and second rare variant with cysteine at position 5 (R _3_C _5_). Synthetic 22-mer peptides representing these three, full-length, signal peptides were tested for their ability to bind Lkt. The synthetic peptides were pre-incubated with *M. haemolytica* Lkt preparations to allow binding, and applied to Lkt-sensitive BL3 cell cultures *in vitro* ( Figure 4A). The common R _3_R _5_ signal peptide was used as a reference since it has a frequency of approximately 0.98 in U.S. cattle, and is predicted to be present on 10 of the full-length CD18 sequences. The synthetic R _3_R _5_ signal peptide had a IC50 of 17.9 μM, which represents the concentration of peptide needed to block 50% of Lkt-induced cytolysis of BL3 cells. The rare H _3_R _5_ signal peptide variant, which is only found on one full-length CD18 variant ( Figure 1C, variant 7), was similar to the reference, with an IC50 of 21.6 μM. In contrast, the rare R _3_C _5_ signal peptide found on full-length CD18 variants 9, 11, 13, and 15 had an IC50 of 5.9 μM, which was 3-fold lower that the reference, indicating an increased affinity for Lkt ( Figure 4A).

The optimum blocking of Ltk-induced cytotoxicity has been previously reported to occur with the 13-mer peptide corresponding to CD18 signal peptide residues 5 to 17 ^10^. Thus, we tested the effect of cysteine at position 5 (C _5_) in this shorter peptide. The synthetic 13-mer C _5_ peptide was 3.6-fold more effective at blocking Lkt toxicity compared to the 22-mer R _3_C _5_ peptide (IC50 of 1.6 and 5.9 μM, respectively). When compared to the 13-mer reference peptide with arginine at position five (R _5_), the 13-mer C _5_ peptide was 8-fold better at blocking Lkt-induced cytotoxicity (IC50 13.0 and 1.6 μM, respectively; Figure 4B). A negative control 13-mer peptide containing randomly assorted amino acids from the reference peptide sequence failed to inhibit Lkt-induced cytolysis, even at the highest concentration tested (50 μM), indicating that inhibition of cytolysis was sequence specific ( Extended Data File S2) ^48^. Together, these results suggest that peptide sequence and length affect Lkt binding.

### Synthetic C _5_ variant CD18 signal peptide blocks Lkt-induced cytolysis of primary bovine cells

The 13-mer C _5_ synthetic signal peptide containing CD18 residues 5 to 17 were also tested for their ability to inhibit Lkt-induced cytolysis of primary cells isolated from cattle. The purpose was to demonstrate that the reduction in cytotoxicity observed with C _5_ signal peptides was comparable between the immortalized cell line and freshly isolated leukocytes from cattle. Like the BL3 cell line, the animals used as donor were homozygous for CD18 variant “1”, and thus have the reference (R _3_R _5_) signal peptide. With primary PBMC ( Figure 5A) or PMN ( Figure 5B), the 13-mer C _5_ synthetic signal peptide was significantly better at blocking Lkt-induced cytotoxicity compared to the R _5_ signal peptide (7- and 14-fold respectively) and was similar to that for BL3 cells (8-fold).

### Arginine at position 4 (R _4_) in the eland CD18 signal peptide disrupts the enhanced Lkt binding attributed by C _5_

**Figure 6.**
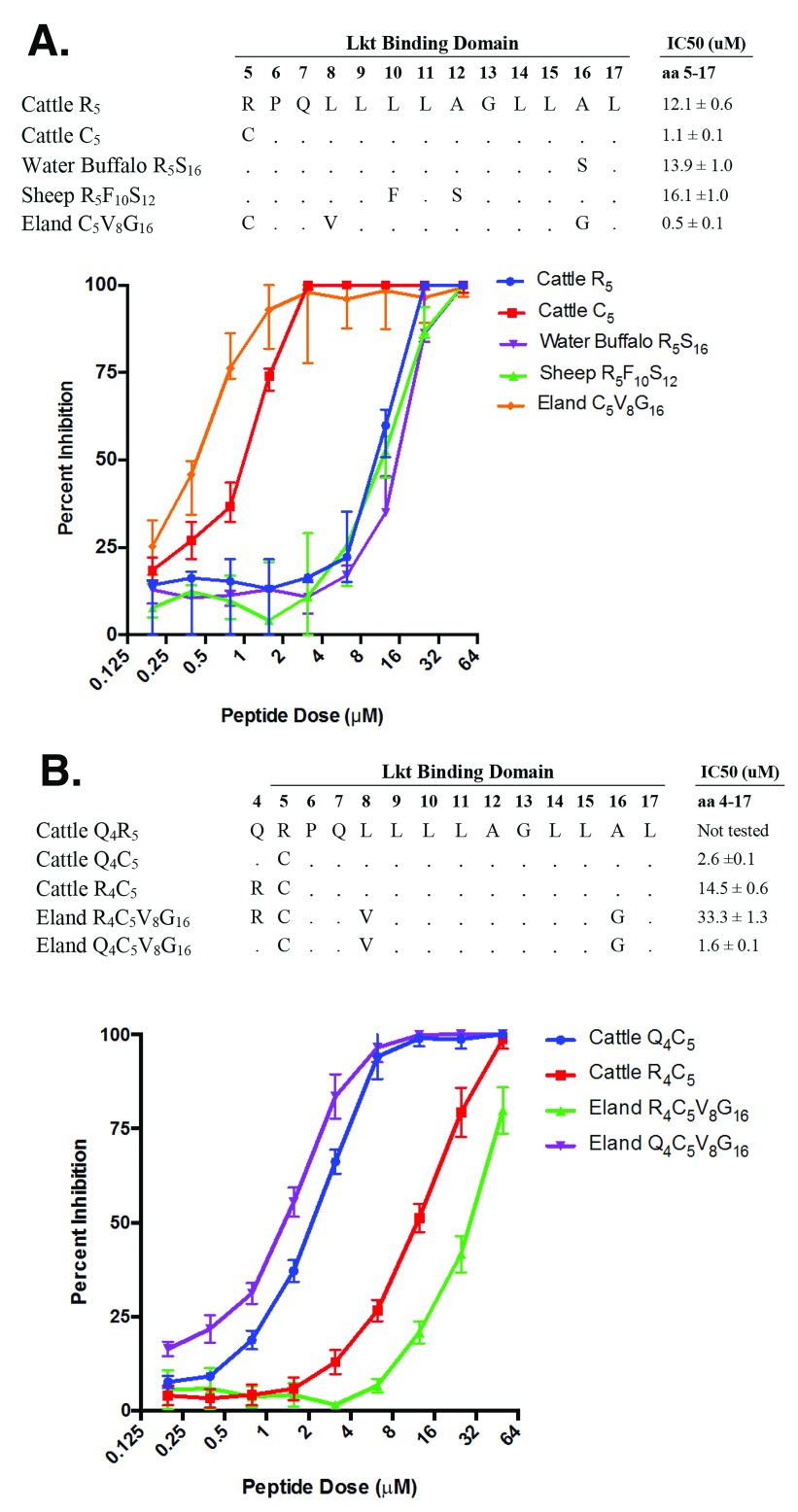
CD18 signal peptides from related mammalian species differentially inhibit leukotoxin (Lkt)-induced cytolysis of bovine BL3 cells. ( **A**) Synthetic 13-mer signal peptides representing amino acids 5 to 17 of sheep, water buffalo, and eland were compared to cattle variants R _5_ and C _5_ for their ability to inhibit Lkt-induced cytolysis of bovine BL3 cells. Peptides were tested using 2-fold dilutions at concentrations ranging from 50 μM to 0.195 μM. ( **B**) Synthetic 14-mer signal peptides representing CD18 amino acids 4 to 17 from eland and cattle variant C _5_ were tested for their ability to bind Lkt. In addition, eland CD18 signal peptides were synthesized where the amino acid at position 4 in eland (arginine, R) was replaced with the amino acid normally found in cattle at this position (glutamine, Q; Eland Q _4_C _5_V _8_G _16_). Similarly, cattle variant C _5_ peptides were synthesized where the amino acid at position 4 in cattle was replaced with the amino acid naturally encoded in eland (Cattle R _4_C _5_). The half-maximal inhibitory concentration (IC50) for each peptide was determined using non-linear regression analyses. Data are expressed as the mean with standard deviation (n=3 or 4).

Some bovid species have CD18 signal peptide sequences that are slightly different from those in cattle ( Figure 6A and Table S3). In water buffalo, the 13-mer peptide sequence corresponding to CD18 signal peptide amino acids 5 to 17 differs at position 16 (S _16_), while the same region in sheep differs at positions 10 and 12 (F _10_S _12_). However, synthetic peptides corresponding to the water buffalo and sheep sequences were similar to the R _5_ reference cattle signal peptide when tested for their ability to bind Lkt and inhibit cytolysis of BL3 cells *in vitro*. In contrast, the eland signal peptide was variant at three positions in this region compared to cattle (C _5_V _8_G _16_), and its 13-mer synthetic signal peptide had a 24-fold decrease in IC50 compared to the reference cattle R _5_ signal peptide and a similar IC50 compared to the reference cattle C _5_ signal peptide ( Figure 6A). However, if the synthetic peptide is expanded to include the eland residue at position 4, the results were different. Eland have an arginine residue at position 4 (R _4_) where other ruminants have a glutamine (Q _4_). A synthetic 14-mer signal peptide with R _4_ (i.e., Eland R _4_C _5_V _8_G _16_) caused a 66-fold reduction in the ability of the eland signal peptide to bind Lkt compared to the 13-mer (C _5_V _8_G _16_) as measured by IC50 ( Figure 6B). When R _4_ was replaced with the Q _4_ normally found in cattle and other ruminants (i.e. Eland Q _4_C _5_V _8_G _16_ versus R _4_C _5_V _8_G _16_), Lkt binding was restored. Similarly, when Q _4_ in the cattle signal peptide (Q _4_C _5_) was replaced with R _4_ (R _4_C _5_) there was a significant reduction in the ability of this peptide to bind Lkt (5.6-fold increase in IC50). These results suggest that the amino acid position 4 in the signal peptide can affect Lkt binding and that R _4_ amino acid sequence in eland may disrupt the enhanced Lkt binding conferred by the C _5_ variant residue.

### A truncated CD18 C _5_ signal peptide has high affinity for Lkt

**Figure 7.**
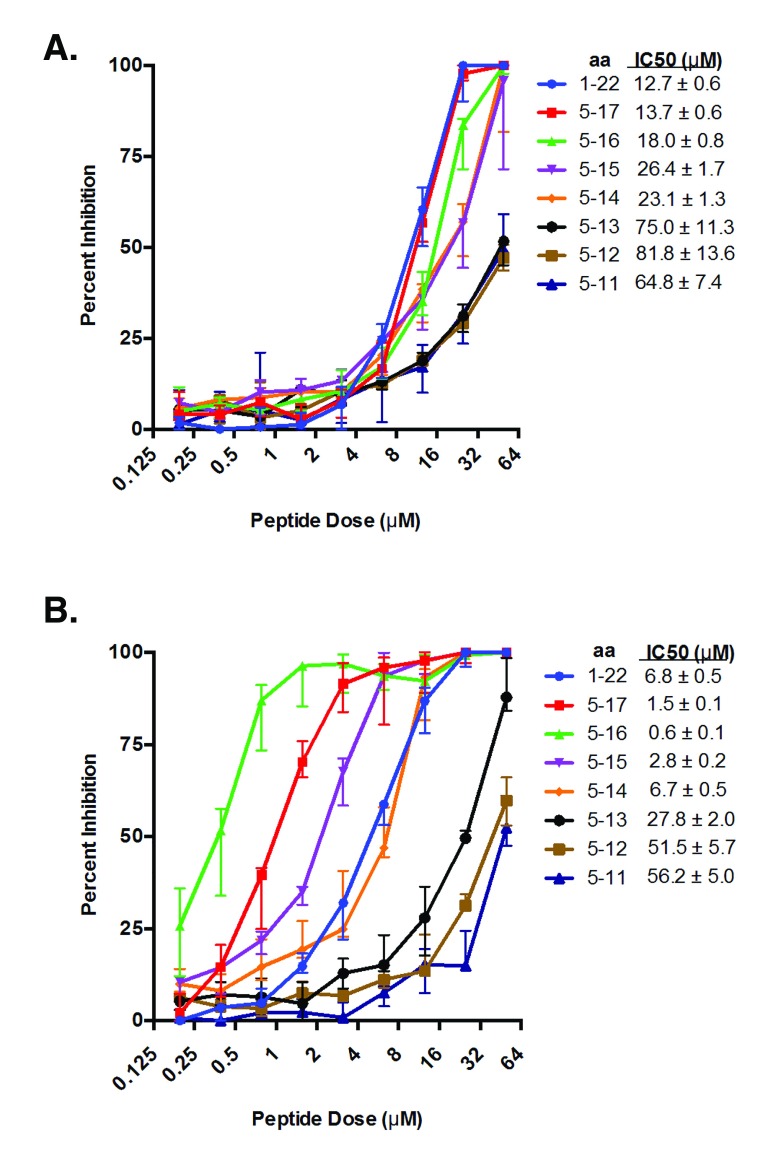
C-terminal truncations reveal the minimal peptide sequence for inhibition of leukotoxin (Lkt)-induced cytolysis. Synthetic CD18 signal peptides were synthesized with single amino acid C-terminal deletions. These peptides were tested for their ability to inhibit Lkt-induced cytolysis of bovine BL3 cells. Peptides were tested at concentrations ranging from 50 μM to 0.195 μM. The half-maximal inhibitory concentration (IC50) for each peptide was determined using non-linear regression analyses. Data are expressed as the mean with standard deviation (n=3).

Toxin inhibitors represent a potentially potent class of therapeutics that could protect animals during acute lung infection. Since truncated synthetic C _5_ signal peptides showed increase affinity for Lkt, various peptide lengths were tested to identify those with maximum Lkt binding. Removing the first four N-terminal amino acids (MLRQ) resulted in no significant difference in Lkt binding for the C _5_ or reference R _5_ signal peptides ( Extended Data Figure S2) ^42^. In contrast, stepwise deletions of C-terminal residues of the C _5_ signal peptide had a significant impact on Lkt binding with the highest affinity being a 12-mer C _5_ signal peptide with amino acids 5 to 16. The IC50 of this 12-mer C _5_ peptide (CPQLLLLAGLLA) was 23-fold lower than the 13-mer reference R _5_ signal peptide (residues 5 to 17) and 30-fold lower than the 12-mer R _5_ signal peptide. Thus, the 12-mer C _5_ peptide (CPQLLLLAGLLA) represents the minimal naturally-occurring peptide sequence with maximal inhibition of Lkt-induced BL3 cell lysis ( Figure 7).

## Discussion

The present report describes bovine CD18 amino acid sequence differences encoded by *ITGB2* in 46 breeds of beef and dairy cattle. All of the protein coding variants discovered were missense mutations and their haplotypes were predicted to encode 15 distinct polypeptide sequences. A C _5_ variant in the CD18 signal peptide region was shown to cause increased binding to *M. haemolytica* Lkt, a secreted toxin that causes cell lysis and acute inflammation leading to lung injury characteristic of bovine respiratory disease. The C _5_ signal peptide variant increased the affinity for Lkt, and this effect was influenced by variation at adjacent residues. The increased Lkt binding and protection from cytotoxic effects were observed in the immortalized BL3 cell line, and freshly isolated PBMC and PMN from beef cattle.

The identification of naturally-occurring CD18 variants with increased binding to Lkt has important potential implications for animal health. For example, cattle with the CD18 C _5_ signal peptide variant may be at increased risk for toxin-related respiratory disease. However, despite the fact that the C _5_ variant was found in four predicted protein variants in taurine and indicine cattle, its overall frequency in the U.S. cattle population in still very low (0.01). Thus, identifying available homozygous cattle for testing their leukocytes *ex vivo* for altered Lkt sensitivity will be challenging. Determining whether this altered binding phenotype contributes to differences in lung lesion severity or disease outcome following *M. haemolytica* infection will also be difficult. Together, these factors suggest that identifying and removing animals with the CD18 C _5_ signal peptide would be premature and unwarranted for most cattle operations at this time.

Recent examples of gene editing in animals have shown that this can be a successful strategy for creating novel host genetic resistance. Groundbreaking work with porcine reproductive and respiratory syndrome virus (PRRSV) has shown that gene editing of a critical entry factor (CD163) confers complete resistance to infection in pigs ^49, 50^. Similarly, genetic resistance to *M. haemolytica* Lkt has been demonstrated in leukocytes isolated from a homozygous, gene-edited, bovine fetus expressing a cleavable CD18 signal peptide ^15^. However, the uncleaved CD18 signal peptide is universally conserved in ruminants and thus, its removal may have deleterious effects on the animal. CD18 forms heterodimers with distinct, but structurally homologous alpha integrin subunits (e.g., CD11a/CD18, CD11b/CD18, and CD11c/CD18), and thus the effect of a cleaved signal peptide may have unknown, but far-reaching effects on biological functions. To date there are no reports of a healthy, live calf expressing a cleavable CD18 signal peptide. Thus, modifying the amino acid sequence of an uncleaved ruminant CD18 signal peptide may be useful as an alternative strategy to reduce Lkt binding, while preserving its normal evolutionarily conserved cellular function.

Identifying the naturally-ocurring CD18 signal peptide residues that influence Lkt binding provides a guide for more extensive analyses that may inform gene edits. Previously, synthetic signal peptides containing the R _5_ residue were used to identify a 13-amino acid minimum CD18 binding site for Lkt (spanning residues 5 through 17, ^10^). Using synthetic peptides representing cattle Q _4_C _5_ and eland R _4_C _5_, we showed that residues in position 4 can drastically affect Lkt binding and that the net charge of the signal peptide is important. The positively charged side chain of R _4_ apparently disrupts the enhanced binding of C _5_ signal peptides compared to the neutral side chain of Q _4_. In addition, the most common CD18 22-mer signal peptide (R _3_R _5_) and the rare H _3_R _5_ signal peptide have a net charge of +2 and reduced Lkt binding compared the R _3_C _5_ peptide (net charge of +1). It would be of interest to determine whether increasing the signal peptide net charge to +3 by introducing another positively charged residue could further reduce Lkt binding. However, there are numerous combinatorial peptide possibilities that could lead to a significantly reduced affinity of CD18 for Lkt, while only a few may preserve the basic biological functions of the signal peptide. Although large-scale screening of candidate peptides *in vitro* and testing them *in vivo* is beyond the scope of this study, our results suggest this as a possible future avenue of research.

An alternative strategy for reducing the impact of Lkt would be to block its activity *in vivo* with synthetic CD18 “decoy” peptides such as the 12-mer C _5_ signal peptide identified here. A similar strategy has been used to neutralize anthrax toxin from *Bacillus anthracis* (reviewed in 51). A synthetic 12-mer peptide antitoxin, attached to liposome scaffolds in multiple copies, protected host cells from cytotoxicity *in vitro* and protected rats from becoming moribund *in vivo* ^52^. Although the feasibility of delivering decoy peptides is unknown in cattle, it could be used to neutralize Lkt, and thus protect calves from the major virulence factor associated with lung pathology in bovine respiratory disease complex. One can further imagine combining decoy peptide technology with gene editing to have alveolar leukocytes secrete decoy peptide inhibitors of Lkt at the sites of *M. haemolytica* infection. Although the theoretical possibilities of using antitoxin and gene editing strategies may be vast, the feasibility of these technologies are still relatively unknown. A better understanding of the underlying molecular mechanisms involved, together with significant improvements in livestock gene editing and decoy peptide technologies, are needed to move the field forward.

## Conclusion

There are more than a dozen missense variants in the CD18 polypeptide, including a C _5_ variant in the signal peptide that affects Lkt binding. Results *in vitro* suggest that animals carrying the C _5_ allele may be more susceptible to the effects of Lkt. These results also identify a potentially potent class of non-antibiotic Lkt inhibitors that could protect cattle from cytotoxic effects during acute lung infections.

## Data availability

### Underlying data

Whole genome sequence files (FASTQ) for the BL3 cell line are available in the NCBI SRA under accession number SRX4645762.

The BL3 sequence data have also been deposited with links to BioProject accession number PRJNA325058 (BioSample SAMN05217649) in the NCBI BioProject database.

In addition, access to the aligned sequences is available via the USDA internet site: https://www.ars.usda.gov/plains-area/clay-center-ne/marc/wgs/celllines/ as described in the Methods.

### Extended data

**Table S1. MALDI-TOF MS assay design for 14 *ITGB2* missense SNPs.** DOI: https://doi.org/10.6084/m9.figshare.7449374.v1 ^53^.

**Table S2. *ITGB2* genotypes recorded manually from WGS reads mapped to UMDv3.1 assembly for the USMARC Beef Cattle Diversity Panel v2.9.** DOI: https://doi.org/10.6084/m9.figshare.7449989.v1 ^45^.

**Table S3. Haplotype-phased genotypes (diplotypes) for *ITGB2* from MALDI-TOF MS assays for 1142 cattle.** DOI: https://doi.org/10.6084/m9.figshare.7450673.v1 ^43^.

**Table S4. Alignment of CD18 sequences from Bilateria species.** DOI: https://doi.org/10.6084/m9.figshare.7450796.v1 ^44^.

**Figure S1. Screen image of Integrated Genome Viewer software displaying *ITGB2* N27KI genotype data for eight bulls.** DOI: https://doi.org/10.6084/m9.figshare.7450814.v1 ^35^.

**Figure S2. Effect of N-terminal truncations of synthetic CD18 signal peptides on Lkt binding.** Synthetic CD18 signal peptides were synthesized with four amino acids removed from N-terminus (MLRQ). The common R _3_R _5_ (A) or the rare R _3_C _5_ variant (B) signal peptides were tested for their ability to inhibit leukotoxin-induced cytolysis of bovine BL3 cells using a MTT cytotoxicity assay. Peptides were tested at concentrations ranging from 50 μM to 0.195 μM. Data are expressed as the mean with standard deviation (n=3). DOI: https://doi.org/10.6084/m9.figshare.7450823.v1 ^42^.

**File S1. VCF file with SNPs from BL3 WGS aligned to the bovine UMD3.1 reference assembly.** https://doi.org/10.6084/m9.figshare.7450826.v1 ^32^.

**File S2. MTT dye-reduction cytotoxicity assay results. Percent inhibition of cytotoxicity was calculated as described in the Methods.** Shown are the results used for statistical analyses of data and for generating Figure 1– Figure 3, Figure 7. DOI: https://doi.org/10.6084/m9.figshare.7451039 ^48^.

## References

[1] Griffin D: Bovine pasteurellosis and other bacterial infections of the respiratory tract. *Vet Clin North Am Food Anim Pract.* 2010;26(1):57–71, table of contents. 10.1016/j.cvfa.2009.10.010 20117542

[2] Whiteley LO Maheswaran SK Weiss DJ: Pasteurella haemolytica A1 and bovine respiratory disease: pathogenesis. *J Vet Intern Med.* 1992;6(1):11–22. 10.1111/j.1939-1676.1992.tb00980.x 1548621

[3] Frank GH Smith PC: Prevalence of Pasteurella haemolytica in transported calves. *Am J Vet Res.* 1983;44(6):981–5. 6870030

[4] Angen O Thomsen J Larsen LE: Respiratory disease in calves: microbiological investigations on trans-tracheally aspirated bronchoalveolar fluid and acute phase protein response. *Vet Microbiol.* 2009;137(1–2):165–71. 10.1016/j.vetmic.2008.12.024 19186010 PMC7117372

[5] Griffin D Chengappa MM Kuszak J: Bacterial pathogens of the bovine respiratory disease complex. *Vet Clin North Am Food Anim Pract.* 2010;26(2):381–94. 10.1016/j.cvfa.2010.04.004 20619191

[6] Singh K Ritchey JW Confer AW: *Mannheimia haemolytica*: bacterial-host interactions in bovine pneumonia. *Vet Pathol.* 2011;48(2):338–48. 10.1177/0300985810377182 20685916

[7] Highlander SK Fedorova ND Dusek DM: Inactivation of *Pasteurella* ( *Mannheimia*) *haemolytica* leukotoxin causes partial attenuation of virulence in a calf challenge model. *Infect Immun.* 2000;68(7):3916–22. 10.1128/IAI.68.7.3916-3922.2000 10858203 PMC101667

[8] Tatum FM Briggs RE Sreevatsan SS: Construction of an isogenic leukotoxin deletion mutant of *Pasteurella haemolytica* serotype 1: characterization and virulence. *Microb Pathog.* 1998;24(1):37–46. 10.1006/mpat.1997.0181 9466945

[9] Ackermann MR Brogden KA: Response of the ruminant respiratory tract to *Mannheimia (Pasteurella)* haemolytica. *Microbes Infect.* 2000;2(9):1079–88. 10.1016/S1286-4579(00)01262-4 10967288

[10] Shanthalingam S Srikumaran S: Intact signal peptide of CD18, the beta-subunit of beta _2_-integrins, renders ruminants susceptible to *Mannheimia haemolytica* leukotoxin. *Proc Natl Acad Sci U S A.* 2009;106(36):15448–53. 10.1073/pnas.0906775106 19706410 PMC2741271

[11] Dassanayake RP Maheswaran SK Srikumaran S: Monomeric expression of bovine beta _2_-integrin subunits reveals their role in *Mannheimia haemolytica* leukotoxin-induced biological effects. *Infect Immun.* 2007;75(10):5004–10. 10.1128/IAI.00808-07 17698568 PMC2044532

[12] Deshpande MS Ambagala TC Ambagala AP: Bovine CD18 is necessary and sufficient to mediate *Mannheimia* ( *Pasteurella*) *haemolytica* leukotoxin-induced cytolysis. *Infect Immun.* 2002;70(9):5058–64. 10.1128/IAI.70.9.5058-5068.2002 12183553 PMC128227

[13] Jeyaseelan S Sreevatsan S Maheswaran SK: Role of *Mannheimia haemolytica* leukotoxin in the pathogenesis of bovine pneumonic pasteurellosis. *Anim Health Res Rev.* 2002;3(2):69–82. 10.1079/AHRR200242 12665107

[14] Slocombe RF Malark J Ingersoll R: Importance of neutrophils in the pathogenesis of acute pneumonic pasteurellosis in calves. *Am J Vet Res.* 1985;46(11):2253–8. 4073635

[15] Shanthalingam S Tibary A Beever JE: Precise gene editing paves the way for derivation of *Mannheimia haemolytica* leukotoxin-resistant cattle. *Proc Natl Acad Sci U S A.* 2016;113(46):13186–13190. 10.1073/pnas.1613428113 27799556 PMC5135351

[16] Shuster DE Bosworth BT Kehrli ME Jr: Sequence of the bovine CD18-encoding cDNA: comparison with the human and murine glycoproteins. *Gene.* 1992;114(2):267–71. 10.1016/0378-1119(92)90586-E 1351021

[17] Shuster DE Kehrli ME Jr Ackermann MR: Identification and prevalence of a genetic defect that causes leukocyte adhesion deficiency in Holstein cattle. *Proc Natl Acad Sci U S A.* 1992;89(19):9225–9. 10.1073/pnas.89.19.9225 1384046 PMC50098

[18] Jeyaseelan S Hsuan SL Kannan MS: Lymphocyte function-associated antigen 1 is a receptor for *Pasteurella haemolytica* leukotoxin in bovine leukocytes. *Infect Immun.* 2000;68(1):72–9. 10.1128/IAI.68.1.72-79.2000 10603370 PMC97103

[19] Ackermann MR Kehrli ME Jr Hawkins HK: Identification of beta _2_ integrins in bovine neutrophils by scanning electron microscopy in the backscatter mode and transmission electron microscopy. *Vet Pathol.* 1993;30(3):296–8. 10.1177/030098589303000311 8101403

[20] Heaton MP Smith TP Carnahan JK: Using diverse U.S. beef cattle genomes to identify missense mutations in *EPAS1*, a gene associated with pulmonary hypertension [version 2; referees: 2 approved]. *F1000Res.* 2016;5:2003. 10.12688/f1000research.9254.2 27746904 PMC5040160

[21] Heaton MP Chitko-McKnown CG Grosse WM: Interleukin-8 haplotype structure from nucleotide sequence variation in commercial populations of U.S. beef cattle. *Mamm Genome.* 2001;12(3):219–26. 10.1007/s003350010269 11252171

[22] Heaton MP Keele JW Harhay GP: Prevalence of the prion protein gene E211K variant in U.S. cattle. *BMC Vet Res.* 2008;4:25. 10.1186/1746-6148-4-25 18625065 PMC2478677

[23] Heaton MP Grosse WM Kappes SM: Estimation of DNA sequence diversity in bovine cytokine genes. *Mamm Genome.* 2001;12(1):32–7. 10.1007/s003350010223 11178741

[24] Nickerson DA Tobe VO Taylor SL: PolyPhred: automating the detection and genotyping of single nucleotide substitutions using fluorescence-based resequencing. *Nucleic Acids Res.* 1997;25(14):2745–51. 10.1093/nar/25.14.2745 9207020 PMC146817

[25] Ewing B Green P: Base-calling of automated sequencer traces using *phred*. II. Error probabilities. *Genome Res.* 1998;8(3):186–94. 10.1101/gr.8.3.186 9521922

[26] Ewing B Hillier L Wendl MC: Base-calling of automated sequencer traces using *phred*. I. Accuracy assessment. *Genome Res.* 1998;8(3):175–85. 10.1101/gr.8.3.175 9521921

[27] Gordon D Abajian C Green P: *Consed*: a graphical tool for sequence finishing. *Genome Res.* 1998;8(3):195–202. 10.1101/gr.8.3.195 9521923

[28] Zimin AV Delcher AL Florea L: A whole-genome assembly of the domestic cow, *Bos taurus*. *Genome Biol.* 2009;10(4):R42. 10.1186/gb-2009-10-4-r42 19393038 PMC2688933

[29] Li H Durbin R: Fast and accurate long-read alignment with Burrows-Wheeler transform. *Bioinformatics.* 2010;26(5):589–95. 10.1093/bioinformatics/btp698 20080505 PMC2828108

[30] Li H Handsaker B Wysoker A: The Sequence Alignment/Map format and SAMtools. *Bioinformatics.* 2009;25(16):2078–9. 10.1093/bioinformatics/btp352 19505943 PMC2723002

[31] McKenna A Hanna M Banks E: The Genome Analysis Toolkit: a MapReduce framework for analyzing next-generation DNA sequencing data. *Genome Res.* 2010;20(9):1297–303. 10.1101/gr.107524.110 20644199 PMC2928508

[32] Workman A Heaton M: File S1. VCF file with SNPs from BL3 WGS aligned to the bovine UMD3.1 reference assembly. *figshare.*Fileset.2018.

[33] Robinson JT Thorvaldsdottir H Winckler W: Integrative genomics viewer. *Nat Biotechnol.* 2011;29(1):24–6. 10.1038/nbt.1754 21221095 PMC3346182

[34] Thorvaldsdottir H Robinson JT Mesirov JP: Integrative Genomics Viewer (IGV): high-performance genomics data visualization and exploration. *Brief Bioinform.* 2013;14(2):178–92. 10.1093/bib/bbs017 22517427 PMC3603213

[35] Workman A Heaton M: Figure S1. Screen image of Integrated Genome Viewer (IGV) software displaying ITGB2 N27KI genotype data for eight bulls. *figshare.*Figure.2018.

[36] Heaton MP Harhay GP Smith TP: Complete Closed Genome Sequences of a *Mannheimia haemolytica* Serotype A1 Leukotoxin Deletion Mutant and Its Wild-Type Parent Strain. *Genome Announc.* 2015;3(3): pii: e00417-15. 10.1128/genomeA.00417-15 25953160 PMC4424311

[37] Murphy GL Whitworth LC Clinkenbeard KD: Hemolytic activity of the Pasteurella haemolytica leukotoxin. *Infect Immun.* 1995;63(8):3209–12. 7622250 10.1128/iai.63.8.3209-3212.1995 PMC173439

[38] Harhay GP Koren S Phillippy AM: Complete Closed Genome Sequences of *Mannheimia haemolytica* Serotypes A1 and A6, Isolated from Cattle. *Genome Announc.* 2013;1(3): pii: e00188-13. 10.1128/genomeA.00188-13 23682137 PMC3656199

[39] van Rensburg E du Preez JC: Effect of pH, temperature and nutrient limitations on growth and leukotoxin production by *Mannheimia haemolytica* in batch and continuous culture. *J Appl Microbiol.* 2007;102(5):1273–82. 10.1111/j.1365-2672.2006.03205.x 17448162

[40] Schneider CA Rasband WS Eliceiri KW: NIH Image to ImageJ: 25 years of image analysis. *Nat Methods.* 2012;9(7):671–5. 10.1038/nmeth.2089 22930834 PMC5554542

[41] Chitko-McKown CG Fox JM Miller LC: Gene expression profiling of bovine macrophages in response to *Escherichia coli* O157:H7 lipopolysaccharide. *Dev Comp Immunol.* 2004;28(6):635–45. 10.1016/j.dci.2003.10.002 15177116

[42] Workman A Heaton M: Figure S2. Effect of N-terminal truncations of synthetic CD18 signal peptides on Lkt binding. *figshare.*Figure.2018.

[43] Workman A Heaton M: Table S3. Haplotype-phased genotypes (diplotypes) for ITGB2 from MALDI-TOF MS assays for 1142 cattle. *figshare.*Dataset.2018.

[44] Workman A Heaton M: Table S4. Alignment of CD18 sequences from Bilateria species. *figshare.*Dataset.2018.

[45] Workman A Heaton M: Table S2. ITGB2 genotypes recorded manually from WGS reads mapped to UMDv3.1 assembly for the USMARC Beef Cattle Diversity Panel v2.9. *figshare.*Dataset.2018.

[46] Nomenclature for incompletely specified bases in nucleic acid sequences. Recommendations 1984. Nomenclature Committee of the International Union of Biochemistry (NC-IUB). *Proc Natl Acad Sci U S A.* 1986;83(1):4–8. 10.1073/pnas.83.1.4 2417239 PMC322779

[47] Hedges SB Marin J Suleski M: Tree of life reveals clock-like speciation and diversification. *Mol Biol Evol.* 2015;32(4):835–45. 10.1093/molbev/msv037 25739733 PMC4379413

[48] Workman A Heaton M: File S1. VCF file with SNPs from BL3 WGS aligned to the bovine UMD3.1 reference assembly. *figshare.*Fileset.2018.

[49] Burkard C Lillico SG Reid E: Precision engineering for PRRSV resistance in pigs: Macrophages from genome edited pigs lacking CD163 SRCR5 domain are fully resistant to both PRRSV genotypes while maintaining biological function. *PLoS Pathog.* 2017;13(2):1006206. 10.1371/journal.ppat.1006206 28231264 PMC5322883

[50] Whitworth KM Rowland RR Ewen CL: Gene-edited pigs are protected from porcine reproductive and respiratory syndrome virus. *Nat Biotechnol.* 2016;34(1):20–2. 10.1038/nbt.3434 26641533

[51] Nestorovich EM Bezrukov SM: Designing inhibitors of anthrax toxin. *Expert Opin Drug Discov.* 2014;9(3):299–318. 10.1517/17460441.2014.877884 24447197 PMC4307821

[52] Basha S Rai P Poon V: Polyvalent inhibitors of anthrax toxin that target host receptors. *Proc Natl Acad Sci U S A.* 2006;103(36):13509–13. 10.1073/pnas.0509870103 16938891 PMC1569193

[53] Workman A Heaton M: Table S1. MALDI-TOF MS assay design for 14 ITGB2 missense SNPs. *figshare.*Dataset.2018.

