# Supplementary material for: A bovine CD18 signal peptide variant with increased binding activity to Mannheimia hemolytica leukotoxin
Source: F1000Res. 2018 Dec 28;7:1985. [Version 1] doi: 10.12688/f1000research.17187.1 (PMC6406179; doi:10.12688/f1000research.17187.1)
Supplement: Peer review history

# Peer review: Reviewer response for version 1

**DOI:** 10.5256/f1000research.18790.r44373

**Published:** 2019-04-09

**Authors:** Jonathan E. Beever

**Affiliations:** 1 University of Illinois at Urbana-Champaign, Urbana, IL, USA; 2 University of Tennessee Institute of Agriculture, Knoxville, TN, USA

This is an excellent manuscript. The work is very well done and the conclusions drawn are well thought out. This manuscript will be well-sited from a scientific standpoint and the results are very translatable.

 Comments:

 In the Abstract -- 'Background' perhaps add bovine as in " ...binds to the CD18  receptor on bovine leukocytes, ..." followed by "Binding of Lkt to bovine CD18 results from an intact 22-amino acid signal peptide which remains uncleaved in all ruminant species." Although these are minor changes, they reflect the knowledge that this binding phenomenon is described specifically in cattle and is now inferred in other susceptible ruminant species based on the Lkt/SD18 signal peptide binding studies in cattle.

 A similar argument could also be applied to the beginning of the second paragraph of the Introduction. However, this paragraph is better in its logical progression.

 It is completely unclear as to why the whole-genome sequencing of the BL3 cell line was performed. Suggest removing that section from the M&M.

I have read this submission. I believe that I have an appropriate level of expertise to confirm that it is of an acceptable scientific standard.

# Peer review: Reviewer response for version 1

**DOI:** 10.5256/f1000research.18790.r44372

**Published:** 2019-03-06

**Authors:** Holly L. Neibergs

**Affiliations:** 1 Department of Animal Sciences, Washington State University, Pullman, WA, USA

The authors have presented a well written article describing a nicely designed and thorough set of experiments to determine if variants in the CD18 signal peptide gene ( *ITGB2*) effect Lkt binding in leukocytes. Lkt is a protein secreted by *M. haemolytica* that activates an inflammatory response leading to fibrinous bronchopneumonia in cattle. *M. haemolytica* is an important bacterial pathogen associated with bovine respiratory disease that causes the death of over one million cattle annually in the U.S. alone. This article provides a better understanding of the molecular mechanisms involved in *M. haemolytica* infection that may lead to more effective means to prevent and treat this common disease in cattle.

 Suggested minor edits were very few:

- Page 3, line 1 of paragraph 1; insert “one of” after “Manheimia haemolytica is"
- Page 6, line 10 of paragraph 2; replace “at run” with “and run at” after (Thermo Fisher Scientific)
- Page 6, line 17 of paragraph 3; remove “in” after “times”
- Page 9, line 9 of paragraph 2; I am not sure I would agree that a conserved sequence with less than 20% identity is remarkable

I have read this submission. I believe that I have an appropriate level of expertise to confirm that it is of an acceptable scientific standard.

# Peer review: Reviewer response for version 1

**DOI:** 10.5256/f1000research.18790.r42342

**Published:** 2019-01-08

**Authors:** Alan L. Archibald

**Affiliations:** 1 The Roslin Institute, Royal (Dick) School of Veterinary Studies (R(D)SVS), University of Edinburgh, Midlothian, UK

The authors have provided a clear and thorough description of their analysis of sequence variation in the bovine ITGB2 gene that encodes CD18. They have not only identified and characterised sequence variants, particularly in two exons of interest, but also tested the effect of these variants in a functional assay in respect of binding to Mannheimia hemolytica leukotoxin.

 The methods used are described in exemplary detail.

 There would be value in noting that some of the missense variants that are a key focus of the analyses have been reported by others previously and citing the relevant rs-identifiers:

 R5C rs517776076

 N27K rs521222417

 R120W rs109222573

 R120Q rs522421330

 D128G rs445709131

 Q392K rs469652717

 P656L rs379267066 

 The authors should be encouraged to submit their new variants to the European Variation Archive that now hosts sequence variation data for non-human species now that dbSNP has ceased this service. The authors should not only submit the sequence variants but also the genotype data and frequencies. Two of the variants listed above do not appear to have survived the migration from dbSNP to EVA and may need to be resubmitted (i.e. R120W / rs109222573; Q392K / rs469652717).

 There a few (very few) minor typographical errors:

 5 uM rather than 5 μM on page 4, column 2 line 11

 a missing gap after the comma on page 6, column 2, line 1

 50 ul rather than 50 μl on page 6, column 2, 5 line from the bottom

 It may be appropriate to mention the contribution of Mannheimia hemolytica to "shipping fever" and note the cost of this disease and pathogen to the US cattle industry.

 The authors discuss their results in the context of a report of gene editing the bovine ITGB2 gene. The paper by Shanthalingam et al. 2016 (cited as reference 15 in this manuscript) report that gene editing a single codon in the ITGB2 gene and generation of a fetus carrying the edited yielded cells that were resistant to Mannheimia hemeolytica in an in vitro assay. The current authors correctly note that there are no reports of live born calves carrying this edit in homozygous form and thus the viability of this gene edit for resistance to Mannheimia hemolytica remains unproven. However, none of the natural ITBG2 variants characterised by the authors delivers resistance. As the authors search for variation was thorough and should have identified almost all variants of useful frequency, then designing variant signal peptide sequences that are not cleaved but do not bind the leukotoxin may be the way forward. The authors' discussion of these points could be more explicit.

I have read this submission. I believe that I have an appropriate level of expertise to confirm that it is of an acceptable scientific standard.
